# Is Phylotranscriptomics as Reliable as Phylogenomics?

**DOI:** 10.1093/molbev/msaa181

**Published:** 2020-07-13

**Authors:** Seongmin Cheon, Jianzhi Zhang, Chungoo Park

**Affiliations:** 1 School of Biological Sciences and Technology, Chonnam National University, Gwangju, Republic of Korea; 2 Department of Ecology and Evolutionary Biology, University of Michigan, Ann Arbor, MI

**Keywords:** phylogenetics, mammals, plants, orthologous genes, evolution

## Abstract

Phylogenomics, the study of phylogenetic relationships among taxa based on their genome sequences, has emerged as the preferred phylogenetic method because of the wealth of phylogenetic information contained in genome sequences. Genome sequencing, however, can be prohibitively expensive, especially for taxa with huge genomes and when many taxa need sequencing. Consequently, the less costly phylotranscriptomics has seen an increased use in recent years. Phylotranscriptomics reconstructs phylogenies using DNA sequences derived from transcriptomes, which are often orders of magnitude smaller than genomes. However, in the absence of corresponding genome sequences, comparative analyses of transcriptomes can be challenging and it is unclear whether phylotranscriptomics is as reliable as phylogenomics. Here, we respectively compare the phylogenomic and phylotranscriptomic trees of 22 mammals and 15 plants that have both sequenced nuclear genomes and publicly available RNA sequencing data from multiple tissues. We found that phylotranscriptomic analysis can be sensitive to orthologous gene identification. When a rigorous method for identifying orthologs is employed, phylogenomic and phylotranscriptomic trees are virtually identical to each other, regardless of the tissue of origin of the transcriptomes and whether the same tissue is used across species. These findings validate phylotranscriptomics, brighten its prospect, and illustrate the criticality of reliable ortholog detection in such practices.

## Introduction

Reconstructing the phylogenetic relationships among various species is a major task of evolutionary biology, because answering almost any evolutionary question requires having a reliable phylogeny of the taxa concerned. Although morphological characters are required in the phylogenetic analysis involving fossils, phylogenies of extant species are now routinely inferred using DNA or protein sequences exclusively. These molecular trees are generally considered more reliable than morphological trees, because there are typically many more molecular characters than morphological characters available for phylogenetic analysis and because homoplasy, which confuses phylogenetic inference, is rarer among molecular characters than morphological characters (Zou and Zhang 2016). However, use of different genes often results in different trees (Rokas et al. 2003), because of sampling error or discordance between gene trees and species trees, among other reasons. Increasing the number of genes and total sequence length in molecular phylogenetics can reduce the sampling error and yield the most common gene tree, which likely represents the species tree. Hence, phylogenomics, inferring phylogenies using genome-scale sequence data, is believed to be a powerful approach to molecular phylogenetics (Eisen and Fraser 2003; Delsuc et al. 2005; Philippe et al. 2005) and has indeed led to a number of well-resolved phylogenies. Nevertheless, despite the drastic cost reduction in DNA sequencing in the last decades, it remains expensive to obtain high-quality genome assemblies with annotations, especially for large eukaryotic genomes (Yandell and Ence 2012; Ekblom and Wolf Jochen 2014) and when many taxa need to be sequenced.

Originally developed for measuring the mRNA concentrations of all expressed genes in a sample (Wang et al. 2009; Martin and Wang 2011), transcriptome sequencing, also known as RNA sequencing (RNA-seq), offers DNA sequences of the transcribed fraction of the genome with a considerably lower cost. The acquisition and use of these DNA sequences for phylogenetics is referred to as phylotranscriptomics, which has been employed by many authors in recent years to resolve the evolutionary relationships of diverse lineages of organisms (Hittinger et al. 2010; Kocot et al. 2011; Smith et al. 2011; Struck et al. 2011; Johnson et al. 2013; Riesgo et al. 2014; Wickett et al. 2014; Zeng et al. 2014; Irisarri et al. 2017; Janouškovec et al. 2017; Price Dana and Bhattacharya 2017). However, whether phylotranscriptomics is as reliable as phylogenomics is unclear, due to several features of phylotranscriptomics that are nonexistent in phylogenomics. First, because not all genes in a genome are expressed in a tissue, transcriptome data do not allow the delineation of the DNA sequences of all genes encoded by a genome. This fact, compounded by gene expression differences among species, makes the identification of orthologous genes from transcriptomes more challenging and less reliable than that from genome sequences. Second, gene expression varies among tissues, and it is unclear whether transcriptomes of certain tissues perform better in phylotranscriptomics than those of other tissues and whether phylotranscriptomics requires using the same tissue from all taxa under consideration. It is obviously difficult to acquire the same tissue from a large number of species. Third, because highly expressed genes, which tend to have slow sequence evolution (Zhang and Yang 2015), are enriched in transcriptomic data, it is unclear whether phylotranscriptomic (PT) results are consequently biased when compared with phylogenomic (PG) results. On the other hand, noncoding regions are likely to be less useful than coding regions for phylogenetics except for closely related species. Hence, transcriptome sequencing is likely more cost-effective than genome sequencing for most phylogenetic tasks. This is especially true if the high cost of genome sequencing limits the sequencing depth and genome assembly quality. Restriction-site-associated DNA sequencing (Andrews et al. 2016), which sequences a faction of each target genome, has also been used for phylogenetics. Because the property of this method is similar to phylogenomics except with smaller data sizes and lower costs (Cariou et al. 2013; Cruaud et al. 2014; Andrews et al. 2016), it will not be considered here.

In this study, we evaluate the performance of phylotranscriptomics with the above questions in mind. Because 1) the true phylogeny of a set of taxa is rarely known, 2) it is hard to simulate transcriptome evolution realistically, and 3) phylotranscriptomics is commonly regarded as an approximation to phylogenomics, we assess the performance of phylotranscriptomics by examining the topological similarity between the transcriptome-based tree and the genome-based tree for the same taxa. Obviously, such analyses require the availability of genome sequences and transcriptome data from the same set of species. Furthermore, the genome sequence-based tree of the taxa should be largely or fully consistent with the commonly accepted evolutionary relationships of the taxa, because otherwise one cannot use the similarity between PT and PG trees to measure the reliability of phylotranscriptomics. Respectively analyzing published data from 22 mammals and 15 plants, we report that, upon rigorous orthologous gene identification, PT trees are virtually identical to PG trees, regardless of the tissue of origin of the transcriptome data and whether the same tissue is used across species.

## Results

### Phylotranscriptomics of 22 Mammals

**Fig. 1.**
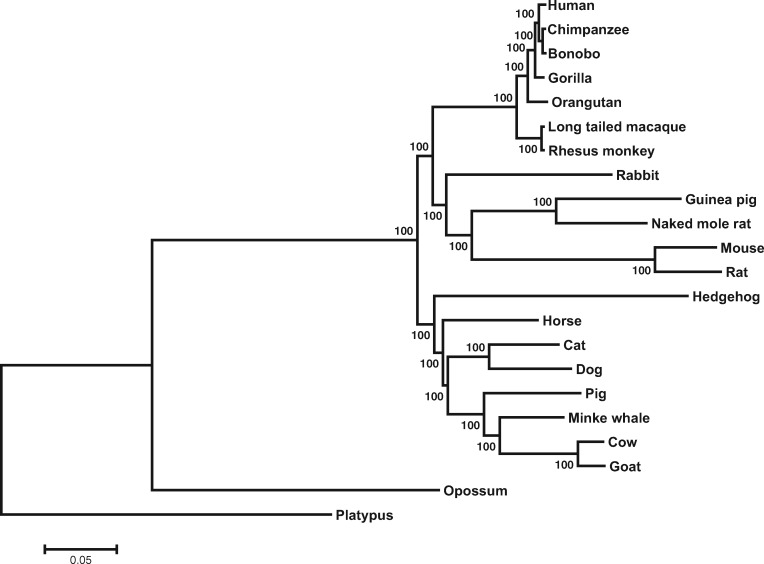
A PG tree of 22 mammals reconstructed using 1,924 one-to-one orthologous genes. Bootstrap percentages estimated from 200 replications are shown on interior branches.

To compare between trees generated using genome and transcriptome data, we selected 22 mammalian species (20 placentals, one marsupial, and one monotreme) with both fully sequenced nuclear genomes and publicly available RNA-seq data from at least three tissues (supplementary table S1, Supplementary Material online). For this comparison to be fair, we should employ the most suitable or widely used computational tools for the PG and PT analyses, respectively. The tools for PG and PT analyses are likely different from each other due to the different types of data used in the two analyses. In the PG analysis, we identified 1,924 one-to-one orthologous genes from the genome sequences using the BlastP-based OrthoMCL method (see Materials and Methods). Upon the alignment of orthologous protein sequences, the alignments were concatenated and gaps removed. We then used RAxML (Stamatakis 2014) to infer the maximum-likelihood tree, which is referred to as the PG tree hereinafter. The PG tree is clearly resolved with each interior branch having a 100% bootstrap support (fig. 1). The topology of the PG tree is largely congruent with previously published molecular trees of these mammals (Miller et al. 2007; Prasad et al. 2008; Morgan et al. 2013).

Transcriptome data from between three and seven of the following seven tissues were publicly available at the time of this study (October 2017) for each of the 22 mammals: brain, kidney, liver, heart, testis, muscle, and lung. In particular, transcriptomes from the first three of these tissues are available for all 22 species. We first analyzed the brain transcriptomes of the 22 mammals in order to build a PT tree. As mentioned, one of the challenges faced by phylotranscriptomics is orthologous gene identification. We thus tested two drastically different methods. The first method, named HaMStR (Ebersberger et al. 2009), is one of the most popular tools for orthologous gene identification from transcriptome data (Kocot et al. 2011; Misof et al. 2014; Zeng et al. 2014). HaMStR combines a profile hidden Markov model (pHMM) search and a subsequent BLAST search to extend existing core orthologs with sequences from further taxa. In the present case, we empirically defined the mammalian core orthologs based on the human, long-tailed macaque, mouse, cow, and dog genome sequences by reciprocal best BLAST hits (see Materials and Methods). The second method, referred to as the YS method (Yang and Smith 2014), builds gene trees from homologous gene sequences in order to identify one-to-one orthologs. Because the YS method uses more stringent criteria than HaMStR in ortholog identification, we expect the YS method to have a higher false negative rate, whereas HaMStR to have a higher false positive rate when compared with each other.

**Fig. 2.**
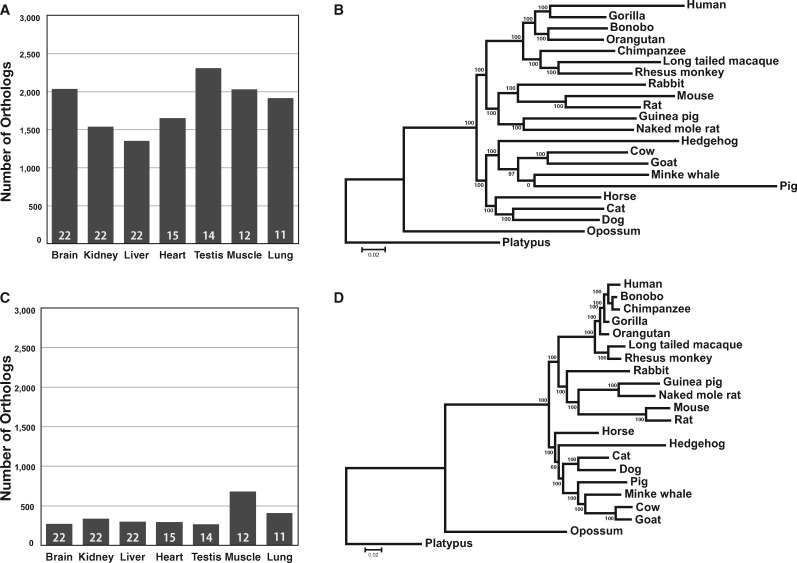
PT trees of mammals inferred from brain transcriptomes. (*A*) Number of one-to-one orthologs identified using HaMStR from the transcriptomes of various tissues. The number in each bar indicates the number of species represented in the transcriptome data. (*B*) The PT tree inferred using 2,035 one-to-one orthologous genes identified by HaMStR from brain transcriptomes. (*C*) Number of one-to-one orthologs identified using the YS method from the transcriptomes of various tissues. The number in each bar indicates the number of species represented in the transcriptome data. (*D*) The PT tree inferred using 270 one-to-one orthologous genes identified by the YS method from brain transcriptomes. Bootstrap percentages estimated from 200 replications are shown on interior branches. The scale bar shows the number of amino acid substitutions per site.

HaMStR identified 2,035 orthologous genes (with trimmed alignments ≥500 codons) from the brain transcriptomes (fig. 2*A*), which allowed the reconstruction of the brain PT tree by RAxML (fig. 2*B*). Although the bootstrap support of the brain PT_HaMStR_ tree is 100% for all but two interior branches, several clades in the tree differ from the corresponding parts of the PG tree and are apparently incorrect. For instance, in the brain PT_HaMStR_ tree, human and gorilla are clustered (with 100% bootstrap support) in exclusion of chimpanzee and bonobo, and whale is grouped with pig (with 0% bootstrap support) instead of cow and goat (fig. 2*B*).

In comparison, the YS method identified only 270 one-to-one orthologous genes (with trimmed alignments ≥150 codons) from the same brain transcriptomes (fig. 2*C*). Yet, the brain PT_YS_ tree (fig. 2*D*) is highly similar to the PG tree in topology, with the only difference being the relative closeness of the hedgehog and horse to the clade including Cetartiodactyla (goat, cow, whale, and pig) and Carnivora (dog and cat). Furthermore, all but one interior branches of the PT tree have 100% bootstrap support.

To examine whether transcriptomes of different tissues yield different PT trees, we similarly analyzed the RNA-seq data from the other six tissues individually. For both HaMStR and YS methods, the number of orthologous genes identified varied among the seven tissues (fig. 2*A* and *C*). This variation is in part due to the inclusion of different numbers of species in the analysis of different tissues. As expected, the number of orthologous genes identified in a tissue tends to decrease with the number of species considered for the tissue, although their correlation is not significant (Spearman’s correlation = −0.41, *P *=* *0.36). The coefficient of variation in orthologous gene number from the HaMStR analysis remains large (0.215) even among the three tissues (brain, kidney, and liver) with data from all 22 species (fig. 2*A*); the corresponding coefficient of variation from the YS results is smaller (0.097) (fig. 2*C*). The seven PT_HaMStR_ trees resulting from the seven tissues (fig. 2*B* and supplementary fig. S1, Supplementary Material online) differ to some extent from one another and from the PG tree when the common species of the trees compared are considered. In comparison, the seven PT_YS_ trees (fig. 2*D* and supplementary fig. S2, Supplementary Material online) look more similar to each other and to the PG tree.

To understand why PT_YS_ trees are more similar than PT_HaMStR_ trees to the PG tree, we benchmarked the orthologs identified in the PG analysis, PT_YS_ analysis, and PT_HaMStR_ analysis against the ortholog annotations in the commonly used OrthoDB. We found that the fraction of incorrectly identified orthologs is the smallest in the PG analysis (1.9%), higher in PT_YS_ (on average 5.5% across the seven tissues), and highest in PT_HaMStR_ (28.0%) (supplementary fig. S3, Supplementary Material online); these differences in error probably cause PT_YS_ to outperform PT_HaMStR_. Furthermore, the alignments of orthologs identified by HaMStR have a high percentage of gap sites when compared with the alignments of orthologs identified by the YS method (supplementary figs. S4 and S5, Supplementary Material online). Interestingly, for an average gene used, the number of species with missing data is lower for HaMStR than YS (supplementary table S2, Supplementary Material online), but this difference may be because of more erroneous orthologs identified by the former than the latter method. Because we required a minimal trimmed alignment length of 500 codons in HaMStR but 150 codons in YS, we wondered whether this difference caused the different performances. We found that the PT_HaMStR_ trees based on orthologous genes with trimmed alignment lengths between 150 and 500 codons (supplementary fig. S6, Supplementary Material online) and those based on orthologous genes with a minimal trimmed alignment length of 500 codons are similarly different from the PG tree (*P *=* *0.13, Mann–Whitney *U* test of equality in topological distances; see below). Thus, the difference in the required minimal alignment length is not the reason why PT_YS_ trees are more similar than PT_HaMStR_ trees to the PG tree.

**Fig. 3.**
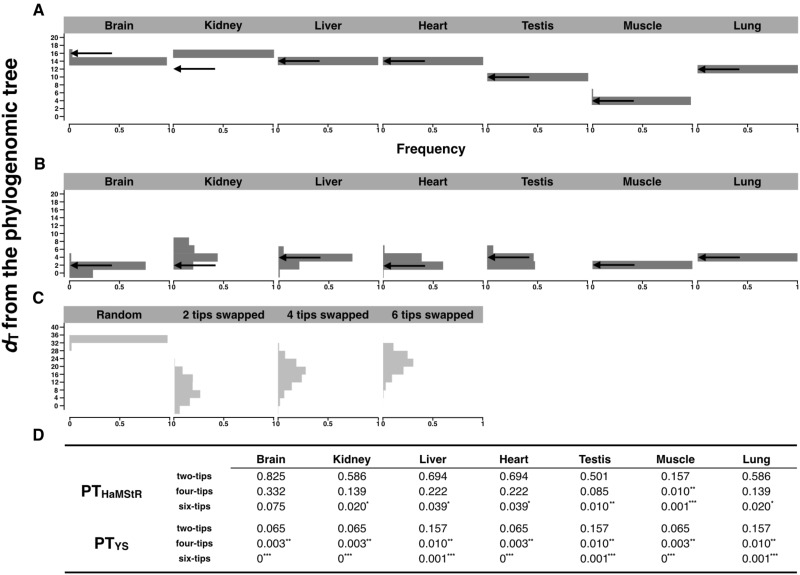
Topological distances (*d*_T_) between the mammalian PG tree and PT trees. (*A*) Distribution of *d*_T_ between the PG tree and 200 bootstrapped PT_HaMStR_ trees constructed using transcriptomes of each tissue. Arrow indicates the result based on the original instead of bootstrapped data. Because different numbers of species are represented in each tissue, one should not directly compare among tissues. (*B*) Distribution of *d*_T_ between the PG tree and 200 bootstrapped PT_YS_ trees constructed using transcriptomes of each tissue. (*C*) Distribution of *d*_T_ between the PG tree and 10,000 random trees of 22 taxa, 10,000 PG trees with two tips swapped, 10,000 PG trees with four tips swapped, and 10,000 PG trees with six tips swapped, respectively. (*D*) Summary of *P* values, which show the probability with which the *d*_T_ between the PG tree and a tip-swapped tree, is equal to or smaller than the observed *d*_T_ between the PG tree and the PT tree being compared. *P <* *0.05, **P <* *0.01, and ***P <* *0.001.

To quantitatively compare PG, PT_YS_, and PT_HaMStR_ trees, we measured the topological distance between a PG and a PT tree from the same set of species by their topological distance *d*_T_, which is twice the number of branch partitions that differ between the two trees (Robinson and Foulds 1981). For every tissue, PT_YS_ has a smaller *d*_T_ than PT_HaMStR_ from the PG tree (fig. 3*A*), demonstrating that, compared with the use of HaMStR, using the YS method in ortholog identification yields PT trees that are more similar to the PG tree (*P *=* *0.016, two-tailed sign test). Furthermore, in all tissues, the distribution of the *d*_T_ values from 200 bootstrap trees is nonoverlapping between the two methods, indicating a significant superiority of the YS method over HaMStR in analyzing these transcriptomes (fig. 3*A* and *B*). Averaged across the seven tissues, *d*_T_ = 3 for PT_YS_ trees (fig. 3*B*), in contrast to 12 for PT_HaMStR_ trees (fig. 3*A*). Hence, the improvement conferred by YS over HaMStR is large. As a comparison, we generated 10,000 random trees among the 22 species and calculated their *d*_T_ from the PG tree. None of these random trees had a *d*_T_ ≤ 16 (fig. 3*C*), which was the maximum *d*_T_ observed for any PT tree, indicating a significantly greater similarity between PT trees and the PG tree than the random expectation.

Another assessment of the topological distance between a PT tree and the corresponding PG tree is to examine whether their *d*_T_ is greater than what is created by swapping two random picked tips (extant taxa) in the PG tree. We generated 10,000 PG trees with two randomly picked tips swapped, 10,000 PG trees with two pairs of randomly selected tips sequentially swapped, and 10,000 PG trees with three pairs of randomly selected tips sequentially swapped, respectively (fig. 3*C*). For the brain transcriptomes, the *d*_T_ between the PG tree and a two-tip-swapped PG tree has a probability of *P*_two-tips_ = 0.825 to be equal to or smaller than the *d*_T_ between the PT_HaMStR_ tree and the PG tree (fig. 3*D*). The *P* value reduces to *P*_four-tips_ = 0.332 when two pairs of tips are swapped and to *P*_six-tips_ = 0.075 when three pairs of tips are swapped (fig. 3*D*). When the PT_YS_ tree instead of the PT_HaMStR_ tree is considered, the above probabilities become *P*_two-tips_ = 0.065, *P*_four-tips_ = 0.003, and *P*_six-tips_ = 0, respectively (fig. 3*D*). This comparison shows that the difference between the PT_HaMStR_ tree and PG tree is equivalent to swaps of one to three pairs of tips, but that between the PT_YS_ tree and the PG tree is no more than the swap of one pair of tips. We performed the same analysis for each of the other tissues, with the consideration of the appropriate PG tree that includes the same species as in the corresponding PT trees. In each tissue, we observed smaller *P* values for the PT_YS_ tree than the PT_HaMStR_ tree (fig. 3*D*). Hence, compared with phylogenomics, YS-based phylotranscriptomics is of similar quality and is much better than HaMStR-based phylotranscriptomics.

In phylotranscriptomics, one can sometimes sample from multiple tissues per species, but our results suggest that, when the YS method is used in ortholog identification, the specific tissue used to profile the transcriptome has only a minimal impact on the tree reconstructed. Specifically, *d*_T_ is between 2 and 4 for the seven tissues considered and is also between 2 and 4 for the three tissues (brain, kidney, and liver) that each has data from all 22 species (fig. 3*B*).

In each of the above PT analyses, the same tissue was used for all species, which may be infeasible under some circumstances. Because nearly one half of all annotated genes of a genome tend to be expressed in a tissue-specific manner (Jongeneel et al. 2005; Whitehead and Crawford 2005; Fagerberg et al. 2014), it is important to investigate whether reliable phylotranscriptomics requires the use of the same tissue from all species concerned. To this end, we randomly chose one transcriptome from each of the 22 species and identified one-to-one orthologous genes from such heterogeneous transcriptomic data using the YS method. This was repeated ten times. On average, we found 171 one-to-one orthologous genes, and the *d*_T_ between a reconstructed PT tree and the PG tree ranges from 2 to 6 with a mean of 4 (supplementary fig. S7, Supplementary Material online), which is only slightly greater than the corresponding *d*_T_ (between 2 and 4 with a mean of 2.7 for the three tissues with all 22 species) when the same tissue is used across all species (fig. 3*B*). This result suggests that, although the use of the same tissue in all species concerned is preferred, using heterogeneous tissues does not substantially reduce the reliability of phylotranscriptomics.

### Phylotranscriptomics of 15 Vascular Plants

**Fig. 4.**
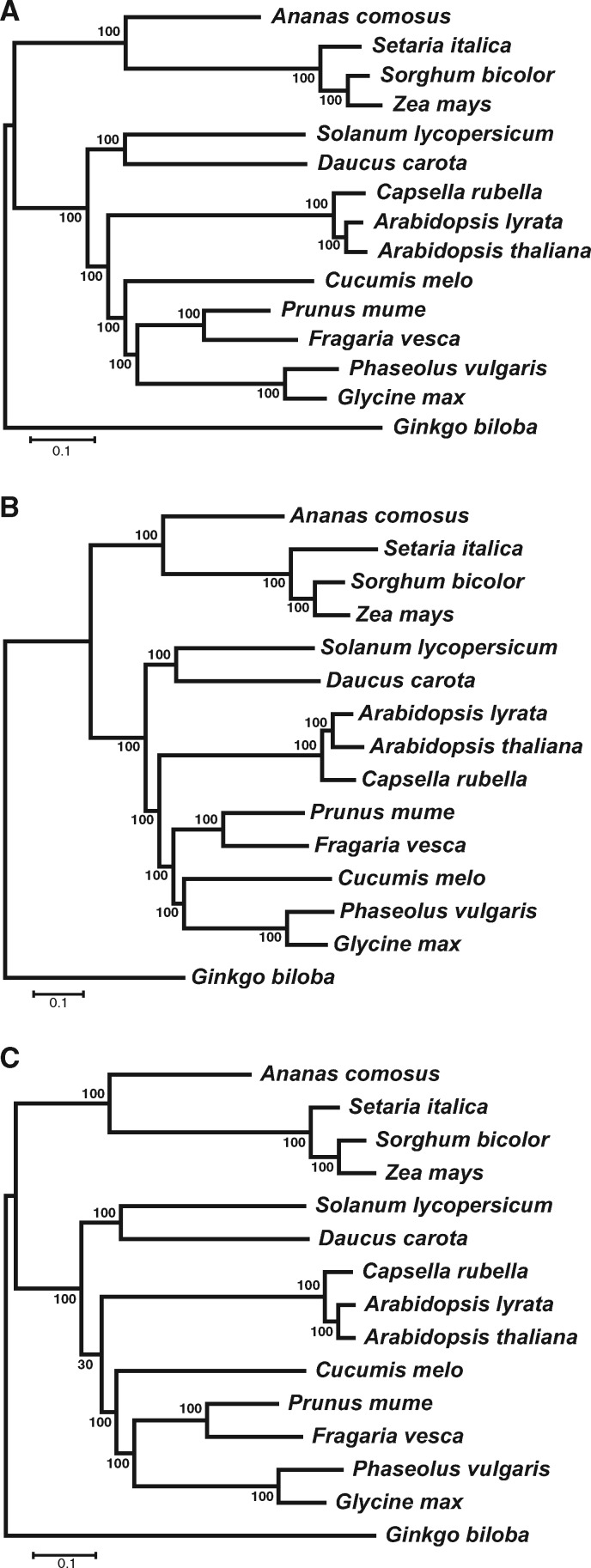
Phylogenetic trees of 15 plants. (*A*) A plant PG tree inferred using 482 one-to-one orthologous genes. (*B*) A plant PT tree inferred using 2,604 one-to-one orthologous genes identified from leaf transcriptomes by HaMStR. (*C*) A plant PT tree inferred using 77 one-to-one orthologous genes identified from leaf transcriptomes by the YS method. Bootstrap percentages estimated from 200 replications are shown on interior branches.

To examine the generality of the results obtained from the 22 mammals, we performed a similar analysis of 15 vascular plants including 14 angiosperms and one gymnosperm (supplementary table S3, Supplementary Material online). Due in a large part to frequent genome duplication followed by gene loss, many plants have unusually dynamic and structurally complex genomes (Coghlan et al. 2005; Tang et al. 2008; Paterson et al. 2010; Jiao et al. 2011), increasing the benefit of phylotranscriptomics over phylogenomics in cost savings. Using orthoMCL, we identified 482 one-to-one orthologous genes (≥150 codons) from the 15 plant genomes. We aligned the corresponding protein sequences and concatenated them before making a maximum-likelihood tree by RAxML. The obtained PG tree is well resolved (fig. 4*A*) and is consistent with the current understanding of plant evolution (Murat et al. 2017).

**Fig. 5.**
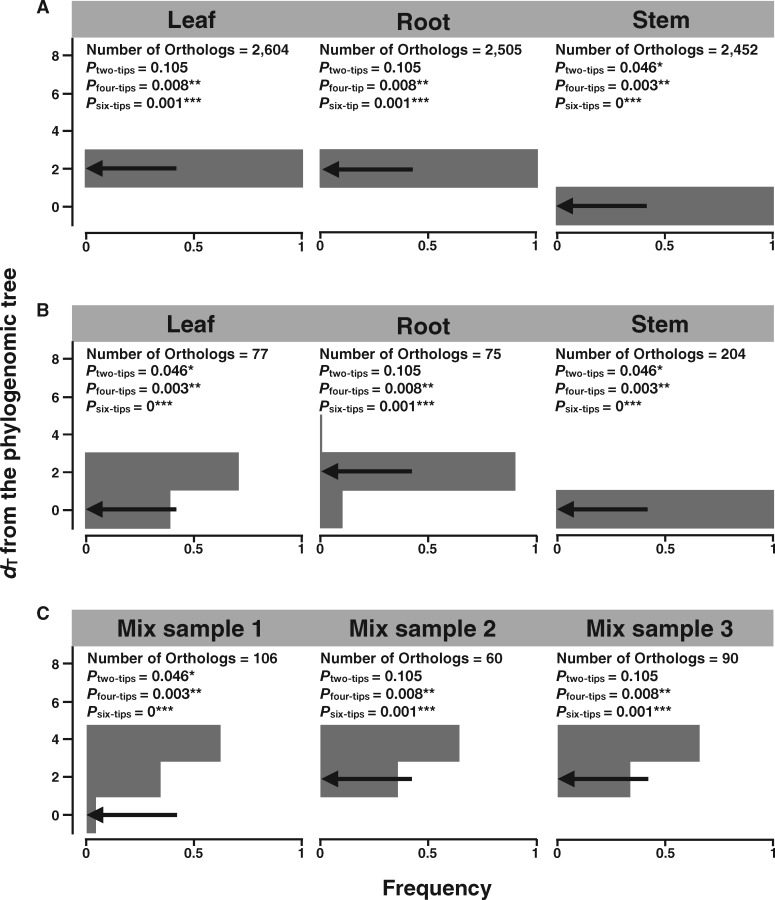
Topological distances (*d*_T_) between the plant PG tree and PT trees. (*A*) Distribution of *d*_T_ between the PG tree and 200 bootstrapped PT_HaMStR_ trees constructed using transcriptomes of each tissue. Because different numbers of species are represented in each tissue, one should not directly compare among tissues. (*B*) Distribution of *d*_T_ between the PG tree and 200 bootstrapped PT_YS_ trees constructed using transcriptomes of each tissue. (*C*) Topological distance (*d*_T_) between the plant PG tree and three PT_YS_ trees inferred using randomly picked transcriptomes from the 15 species. In all panels, arrow indicates *d*_T_ based on the original data whereas the gray shade shows the frequency distribution of *d*_T_ from 200 bootstrapped samples. *P* values show the probability with which the *d*_T_ between the PG tree and a tip-swapped PG tree is equal to or smaller than the observed *d*_T_ between the PG tree and the PT tree. *P <* *0.05, **P <* *0.01, and ***P <* *0.001. Numbers of orthologs used are indicated.

We found publicly available RNA-seq data from three tissues (leave, root, and stem) for each of 11 species and those from two tissues (leave and root) for each of the four remaining species (supplementary table S3, Supplementary Material online). From the three tissues, we identified an average of 2,520 and 119 orthologous genes (≥150 codons) using HaMStR and YS methods, respectively. The same procedure was used to identify orthologs in plants and mammals. Probably because plant genomes experienced genome duplication, the number of one-to-one orthologs identified is considerably fewer in plants (119) than in mammals (270). When the leaf transcriptomes were analyzed, the PT_HaMStR_ tree (fig. 4*B*) has the same topology as the PG tree except for the position of *Cucumis melo*, whereas the PT_YS_ tree (fig. 4*C*) has the same topology as the PG tree. That is, the leaf PT_HaMStR_ (fig. 5*A*) and PT_YS_ (fig. 5*B*) trees have *d*_T_ = 2 and 0 from the PG tree, respectively. When the root transcriptomes were analyzed, both PT_HaMStR_ (supplementary fig. S8*A*, Supplementary Material online) and PT_YS_ (supplementary fig. S9*A*, Supplementary Material online) trees have *d*_T_ = 2 from the PG tree (fig. 5*A* and *B*). When the stem transcriptomes were analyzed, both PT_HaMStR_ (supplementary fig. S8*B*, Supplementary Material online) and PT_YS_ (supplementary fig. S9*B*, Supplementary Material online) trees have *d*_T_ = 0 from the PG tree for the 11 species concerned (fig. 5*A* and *B*). Comparing between PT trees and PG trees with two or four tips swapped showed that PT trees are typically no more different than swapping two tips from the PG tree (fig. 5*A* and *B*). Thus, phylotranscriptomics, especially when the YS method is used for ortholog identification, is almost as reliable as phylogenomics for the 15 vascular plants. When randomly sampling one of the available tissues from each species, we found that the YS-based PT trees are still close to the PG tree, with *d*_T_ = 0 or 2 in three replicates of random sampling (fig. 5*C*).

### Robustness of the Results

To examine whether the above findings in mammals and plants are sensitive to the particular methods or models used, we applied the following five alternative methods or models. First, instead of using the YS method, we used a recently developed tree-based orthology refinement method named PhyloPypruner for identifying orthologs from transcriptomes. We found that PhyloPypruner-based PT trees (supplementary fig. S10, Supplementary Material online) are almost identical to the corresponding PT_YS_ trees and PG trees (supplementary fig. S11, Supplementary Material online). Second, instead of using OrthoMCL, we used OrthoFinder to identify orthologs from genome sequences. We identified 2,397 and 482 one-to-one orthologous genes from the mammalian and plant genomes, respectively. Except for the position of the horse that has a low bootstrap value of 67%, the mammalian and plant PG trees were unaltered by using these orthologs (supplementary fig. S12, Supplementary Material online). In addition, the same method was applied to the mammalian and plant transcriptomes. The *d*_T_ values between the PT trees (supplementary fig. S13, Supplementary Material online) under OrthoFinder and the PG tree were not significantly different (*P *=* *0.58, Mann–Whitney *U* test) from the corresponding original *d*_T_ values based on the YS method. Third, instead of using HaMStR, we used Orthograph to circumvent redundant transcript assignments when identifying orthologs but did not find it to improve phylotranscriptomics. For instance, the brain PT_Orthograph_ tree (supplementary fig. S14, Supplementary Material online) has a *d*_T_ of 26 from the PG tree, even higher than that (16) of the brain PT_HaMStR_ tree. Fourth, instead of using RAxML with the PROTCATWAG model, we used IQ-TREE under the LG + C60 + F + R model to infer the PT trees of mammals and plants. Except for the three tissues (muscle, lung, and root) whose *d*_T_ values decreased from 2 to 0 (muscle and root) or from 4 to 0 (lung), *d*_T_ values between PT and PG trees were unaltered (supplementary figs. S15 and S16, Supplementary Material online). Finally, instead of using the concatenation-based phylogenetic analysis, we tried a coalescent-based analysis (ASTRAL-III) but found the results similar. For instance, from the PG tree, the brain PT_YS_ tree from the coalescent approach (supplementary fig. S17, Supplementary Material online) and that from the concatenation approach (fig. 2*D*) both show a *d*_T_ of 2. Together, these results suggest that our conclusion that phylotranscriptomics is as reliable as phylogenomics is robust.

## Discussion

With the aim of comparing the performance of phylotranscriptomics with that of phylogenomics, we respectively analyzed 22 mammals and 15 vascular plants that have both publicly available genome sequences and RNA-seq data from multiple tissues. We found that when orthologous genes are identified from transcriptomes using the YS method, the inferred PT tree tends to be highly similar to the PG tree, even when the transcriptomic data of different species originate from different tissues. This finding demonstrates that phylotranscriptomics is a good approximation to phylogenomics and alleviates the constraint of sampling the same tissue across a diverse array of species, which may be infeasible under many circumstances. Furthermore, our results imply that when transcriptomes from multiple tissues are available from a species, it is valuable to use the merged transcriptome data of the species in phylotranscriptomics. Given the relatively low cost of transcriptome sequencing compared with genome sequencing, our finding is expected to stimulate wider uses of phylotranscriptomics.

Despite the increasing quantity of genome-scale molecular data, several studies have produced conflicting phylogenetic results even with large molecular data. For example, three PG analyses of early animal diversification yielded conflicting conclusions regarding the origin of metazoa (Dunn et al. 2008; Philippe et al. 2009; Schierwater et al. 2009). A similar situation exists regarding the root of the placental mammal tree (Meredith et al. 2011; McCormack et al. 2012; Song et al. 2012; Leary et al. 2013). Apparently, adopting big data by only increasing the sequence length is not sufficient to resolve some difficult trees. Appropriate and extensive taxon sampling may help (Philippe et al. 2011). The reduction in the cost of sequencing per species when one adopts phylotranscriptomics instead of phylogenomics allows broadening taxon sampling, which would further help resolve some of the most difficult trees. One caveat in the above reasoning is the fact that the number of orthologous genes that can be used for phylogenetic inference is expected to reduce with the number of taxa included. To assess the impact of this problem, we analyzed two subsets of the 22 mammals in figure 1. The first subset contains seven species (human, rabbit, mouse, hedgehog, dog, opossum, and platypus), whereas the second, larger subset contains 14 species (first subset plus orangutan, Rhesus monkey, guinea pig, mole rat, horse, pig, and cow). These subsets are constructed to represent major lineages in the mammalian tree in figure 1 but with different degrees of taxon sampling. We ask whether using the larger subset of 14 species (or the full set of 22 species) is better than using the smaller subset for inferring the phylogenetic relationships of the seven species in the smaller subset. Clearly, using the larger subset or full set increases taxon sampling, but it may reduce the number of usable orthologs. Indeed, when analyzing the brain transcriptomes, we found the number of orthologous genes to reduce from 1991 for the smaller subset to 705 for the larger subset to 235 for the full set, based on the orthologs identified by OrthoFinder. When the phylogenetic relationships of the seven species of the smaller subset are concerned, *d*_T_ of the PT tree from the PG tree is 0 when the smaller subset of transcriptomes are used but becomes 6 and 4 respectively when the larger subset and full set are used. Similar results were observed when kidney or liver transcriptomes were analyzed. Hence, more studies are needed to find the right amount of taxon sampling for reliable PT analysis.

Our study showed that the success of phylotranscriptomics relies on rigorous orthologous gene identification. It is worth noting that we identified orthologs with a gene-tree-free method (orthoMCL) in phylogenomics. Yet, in phylotranscriptomics, the gene-tree-based ortholog identification implemented by Yang and Smith (2014) is superior to the tree-free ortholog identification implemented in the popular HaMStR, despite an order of magnitude fewer orthologs identified by the former than the latter. It is possible that the gene-tree-free ortholog identification is sufficiently accurate in analyzing genome sequences but not so when applied to transcriptomes, because many genes may be missing from the latter due to low expressions. A key parameter in HaMStR is the set of core orthologs, which we defined empirically from five mammalian or plant genomes (see Materials and Methods). To investigate how the core orthologs affect ortholog identification from transcriptomes, we also tried a predefined set of 1,032 eukaryotic core orthologs in the HaMStR model organisms data set, which is the most commonly used set in recent PT analyses. The brain PT_HaMStR_ tree inferred under this set of eukaryotic core orthologs is worse than the brain PT_HaMStR_ tree inferred under 14,018 mammalian core orthologs (supplementary fig. S18*A* and *B*, Supplementary Material online), demonstrating the sensitivity of ortholog identification by HaMStR to the core orthologs used and the benefit of using core orthologs matching the group of species considered. However, using the plant core orthologs versus eukaryotic core orthologs does not affect the HaMStR-based PT tree of the plants (supplementary fig. S18*C* and *D*, Supplementary Material online), probably because the plant data are less sensitive to ortholog identification algorithms. We found that the extent by which PT_YS_ is superior to PT_HaMStR_ is greater for the mammalian data than the plant data. This may be explained by the fact that the fraction of incorrectly identified orthologs is more similar between YS and HaMStR for plants than for mammals (supplementary fig. S3, Supplementary Material online), but the reason behind this plant–mammal disparity is unclear. It is a pleasant surprise that the YS method, developed for plant phylotranscriptomics, is even more useful for animal phylotranscriptomics.

We note, however, that all of our conclusions are based on only two phylogenetic clades: mammals and vascular plants. Although there is no strong reason to suspect otherwise, the generality of these conclusions across the tree of life awaits further exploration.

## Materials and Methods

### Genome and Transcriptome Data

We selected 22 mammals and 15 plants with fully sequenced nuclear genomes and publicly available RNA-seq data. The data sets used are listed in supplementary tables S1 and S3, Supplementary Material online.

### Transcriptome Data Processing

To obtain high-quality clean reads (i.e., excluding adapter sequences, poly-N sequences, or low quality bases), we processed all raw RNA-seq data using Trimmomatic (v0.35) (Bolger et al. 2014). The clean reads from each sample were then used for de novo transcriptome assembly by Trinity (version 2.20) (Haas et al. 2013) with default settings. After assembly, open reading frames (ORFs) were predicted using TransDecoder (version 3.0.0) (https://github.com/TransDecoder/TransDecoder/wiki) assisted by BlastP searches with an *E*-value cutoff of 10^−5^ using UniprotKB/Swiss-Prot database (http://www.uniprot.org). ORFs shorter than 100 codons were discarded. ORF sequences with >99% amino acid sequence identity were clustered using the CD-HIT program (version 4.6.5) (Li and Godzik 2006). We performed a BUSCO analysis (Waterhouse et al. 2018) to evaluate the completeness of each transcriptome we investigated (supplementary fig. S19, Supplementary Material online) but found no clear association between the completeness and the number of orthologous genes or *d*_T_ from the PG tree.

### Identification of Orthologs from Complete Genome Sequences

OrthoMCL (Li et al. 2003) was run in default settings with all-against-all BlastP analysis to identify orthologous proteins in the 22 mammalian genomes. Pairwise sequence similarities between protein sequences were calculated using BlastP with an *E*-value cutoff of 10^−5^. Markov clustering was applied using an inflation parameter of 1.4 to improve sensitivity and specificity. To avoid complications introduced by paralogous genes in PG inference, we excluded orthologous gene groups containing more than one gene from any given species and exclusively selected orthologous genes shared by all 22 mammals to infer the PG tree. The same method was used in the analysis of 15 plant genomes.

We also used OrthoFinder (v2.3.3) (Emms and Kelly 2015) under Diamond (Buchfink et al. 2015) sequence search (v0.9.24.125) with default options for ortholog identification in both mammalian and plant genomes. The above clustering algorithm and the same exclusion criteria for grouping orthologous genes were applied.

### Identification of Orthologs from Transcriptomes

Five methods were employed to identify orthologous protein coding genes from mammalian transcriptomes without the use of genome sequences. First, we employed HaMStR (version 13.2.6) (Ebersberger et al. 2009), which in turn used BlastP (Altschul et al. 1997) and HMMER (Eddy 1998) to search the combined assembled data for protein sequences matching a set of known core orthologs. Two distinct sets of core orthologs were used. The first, referred to as eukaryotic core orthologs, is a predefined set of 1,032 single-copy orthologous genes in the InParanoid database (O’Brien et al. 2005) derived from the genome sequences of *Home sapiens*, *Ciona intestinalis*, *Drosophila melanogaster*, *Caenorhabditis elegans*, and *Saccharomyces cerevisiae*. The second, referred to as the mammalian core orthologs, is a set of 14,018 single-copy orthologous genes identified from the genome sequences of *Homo sapiens*, *Macaca fascicularis*, *Mus musculus*, *Bos taurus*, and *Canis familiaris* using OrthoMCL with BlastP *E*-value cutoff of 10^−5^ and Markov clustering inflation index of 1.4. According to the HaMStR method, our mammalian-translated unigenes were searched from any one of the 1,032 eukaryotic core orthologs (or 14,018 mammalian core orthologs) with pHMMs. The matched unigenes were compared with human proteins (as a reference) using BlastP. If a reciprocal best BLAST hit existed between these genes, the unigene was placed in that orthologous gene. Finally, for phylogenic inference, we kept those orthologous genes with genes present with single-copy (one-to-one) orthologs in at least 50% of the species in the group. The same analyses were conducted for plant transcriptomes, where core orthologous genes were defined using the genome sequences of *Arabidopsis thaliana*, *Zea mays*, *Solanum lycopersicum*, *Phaseolus vulgaris*, and *Ananas comosus* in conjunction with *Arabidopsis thaliana* proteins as a reference.

Second, we applied the gene-tree-based orthology inference method of Yang and Smith (2014). Using all-against-all BlastP comparisons (*E*-value cutoff of 10^−5^ and max_target_seqs 1,000) among a set of protein sequences inferred from mammalian transcriptomes, we carried out initial homology searches. The resulting BlastP hits which had at least 30% aligned regions and at least 30% identical amino acids in the aligned regions, and with a hit fraction being at least 0.3, were retained. To obtain putative homology groups, we performed Markov clustering (MCL) on the filtered all-against-all BlastP results. For further homology inference, sequences shorter than 30 amino acids were excluded, and clusters with at least half of the species represented were retained. The sequences of each resulting cluster were aligned and trimmed using MAFFT (v7.149, options: -genafpair, -maxiterate 1,000) (Katoh and Standley 2013) and Phyutility (v2.2.6, option: -clean 0.1) (Smith and Dunn 2008), respectively. Gene trees were estimated using RAxML (v8.2.9) (Stamatakis 2014) with the model of PROTCATWAG. To prune spurious branches from the input tree, a terminal branch was removed if it was more than ten times the length of its sister branch and was longer than 0.6. Because multiple isoforms of the same gene inferred from transcriptomes could form monophyletic or paraphyletic groups, only the ones that had the most unambiguous characters in the trimmed alignment were retained. Branches longer than 0.5 were excluded to remove deep paralogs. The resulting tree was trimmed to produce one-to-one orthologous genes that were most likely present as single-copy genes in the ancestor of the mammals.

Third, we used another tree-based orthology inference method named PhyloPypruner (v0.8.4) (https://pypi.org/project/phylopypruner/). We reused the input trees and alignments generated by the YS method and implemented PhyloPypruner with default options to prune erroneous branches.

Fourth, we used Orthograph (v0.6.3) (Petersen et al. 2017) to search orthologous genes from transcriptomes. It employs a best reciprocal hit search strategy using pHMMs and maps nucleotide sequences to the globally best matching cluster of the mammalian core orthologs.

Finally, the OrthoFinder (v2.3.3) (Emms and Kelly 2015) used for ortholog identification from genomes was also applied to both mammalian and plant transcriptomes.

### Inference of Phylogenomic and Phylotranscriptomic Trees

Amino acid sequences of orthologous genes were aligned with Prank (http://wasabiapp.org/software/prank/) using default options. The aligned sequences were trimmed using Phyutility (option: -clean 0.3). Any trimmed alignments <500 amino acids were discarded, except in the case of all plant trees and YS-method-based mammalian PT trees where this length cutoff was set at 150. Maximum-likelihood trees based on the concatenated trimmed alignments were inferred by RAxML with the PROTCATWAG model and IQ-TREE (v1.6.10) (Nguyen et al. 2015) under the LG + C60 + F + R model.

### Coalescent-Based Species Tree Reconstruction

We inferred gene trees from individual orthologous groups using IQ-TREE (v1.6.10) (Nguyen et al. 2015) with the LG + C60 + F + R model. A species tree was then reconstructed from the estimated gene trees using ASTRAL-III (version 5.7.3) (Zhang et al. 2018).

### Generation of Random Trees

To simulate random trees with a given number of taxa, we used the *rtree* function in the *ape* package (v5.0) implemented in R (https://cran.r-project.org). In addition, we used in-house Python scripts to construct tip-swapped trees by swapping two randomly selected tips in a tree at a time until the number of predefined iterations was reached.

### Topological Distance between Two Trees

Tree topologies were compared using the R library package *ape* (v5.0) (Popescu et al. 2012) with the function *dist.topo*, which implemented the topological distance (Robinson and Foulds 1981). All statistical analyses were performed using R (R Development Core Team, R Foundation for Statistical Computing, Vienna, Austria).

### Benchmarking Ortholog Identification Methods

To investigate whether the orthologs identified by the HaMStR and YS methods are correct, we used the OrthoDB hierarchical catalogue (version 10) (Kriventseva et al. 2019) as the gold standard. Using BlastP searches (*E*-value cutoff of 10^−5^), each gene in each ortholog group was assigned an OrthoDB ID. If all genes in an ortholog group have the same ID, the group is regarded as having correct orthology. Otherwise, we consider it incorrect if at least one gene in the ortholog group has a different ID, or unannotated if any gene in the group has no OrthoDB ID.

## Supplementary Material

Supplementary data are available at *Molecular Biology and Evolution* online.

## Supplementary Material

msaa181_supplementary_data
