# Supplementary material for: Is Phylotranscriptomics as Reliable as Phylogenomics?
Source: Mol Biol Evol. 2020 Jul 13;37(12):3672–83. doi: 10.1093/molbev/msaa181 (PMC7743905; doi:10.1093/molbev/msaa181)
Supplement: msaa181_supplementary_data [file msaa181_supplementary_data.pdf]

Supplementary materials for "Is phylotranscriptomics as reliable as phylogenomics?"

Supplementary materials include:

Supplementary figure legends

Figures S1-S19

Tables S1-S3

Legends of supplementary figures

Fig. S1. PT trees of mammals inferred from (A) kidney, (B) liver, (C) heart, (D) testis, (E) muscle, and (F) lung transcriptomes upon orthologous gene identification using HaMStR.

Fig. S2. PT trees of mammals inferred from (A) kidney, (B) liver, (C) heart, (D) testis, (E) muscle, and (F) lung transcriptomes upon orthologous gene identification using the YS method.

Fig. S3. Fractions of orthologs identified by the (A) HaMStR and (B) YS methods that are correct, incorrect, or unannotated in OrthoDB.

Fig. S4. Features of multiple sequence alignments of orthologous genes identified by HaMStR from (A) mammalian or (B) plant transcriptomes. The left Y-axis indicates the mean fraction of gap sites in the alignment (represented by green color). The right Y-axis indicates both the mean gapless alignment length per CDS (bp) (represented by blue color) and the number of orthologous genes (represented by red color).

Fig. S5. Quality of multiple sequence alignments of orthologous genes identified by the YS method from (A) mammalian or (B) plant transcriptomes. The left Y-axis indicates the mean fraction of gap sites in the alignment (represented by green color). The right Y-axis indicates both the mean gapless alignment length per CDS (bp) (represented by blue color) and the number of orthologous genes (represented by red color).

Fig. S6. PT trees of mammals inferred from (A) brain, (B) kidney, (C) liver, (D) heart, (E) testis, (F) muscle, and (G) lung transcriptomes upon orthologous gene identification using HaMStR with trimmed alignment lengths between 150 and 500 codons.

Fig. S7. Topological distance (d_T) between the mammalian PG tree and 10 PT_{YS} trees inferred using randomly picked transcriptomes from the 22 species. Arrow indicates d_T based on the original data whereas the gray shade shows the frequency distribution of d_T from 200 bootstrapped samples. *P* values show the probability with which the d_T between the PG tree and

a tip-swapped PG tree is equal to or smaller than the observed d_T between the PG tree and the PT tree. *, $P < 0.05$; **, $P < 0.01$; ***, $P < 0.001$.

Fig. S8. Plant phylotranscriptomic trees inferred using 2,505 and 2,452 one-to-one orthologous genes identified by the HaMStR method from the root (A) and stem (B) transcriptomes, respectively.

Fig. S9. Plant phylotranscriptomic trees inferred using 75 and 204 one-to-one orthologous genes identified by the YS method from the root (A) and stem (B) transcriptomes, respectively.

Fig. S10. PT trees of mammals or plants inferred from (A) brain, (B) kidney, (C) liver, (D) heart, (E) testis, (F) muscle, (G) lung, (H) leaf, (I) root, or (J) stem transcriptomes upon orthologous gene identification using PhyloPypruner.

Fig. S11. Topological distances (d_T) between the PG tree and PT trees in Fig. S9 for (A) mammals or (B) plants. Arrows indicate the results based on the original data whereas shades indicate the results based on 200 bootstrapped datasets.

Fig. S12. PG trees of (A) mammals and (B) plants based on orthologs identified by OrthoFinder.

Fig. S13. PT trees of mammals or plants inferred from (A) brain, (B) kidney, (C) liver, (D) heart, (E) testis, (F) muscle, (G) lung, (H) leaf, (I) root, or (J) stem transcriptomes upon orthologous gene identification using OrthoFinder.

Fig. S14. A mammalian PT tree inferred from brain transcriptomes using orthologs identified by Orthograph.

Fig. S15. PT trees of mammals or plants inferred from (A) brain, (B) kidney, (C) liver, (D) heart, (E) testis, (F) muscle, (G) lung, (H) leaf, (I) root, or (J) stem transcriptomes reconstructed using IQ-TREE with LG+C60+F+R model. Orthologs are identified using the YS method.

Fig. S16. Topological distances (d_T) between the PG trees and PT trees of Fig. S12 for (A) mammals or (B) plants. Arrows indicate the results based on the original data whereas shades indicate the results based on 200 bootstrapped datasets.

Fig. S17. A phylotranscriptomic tree of mammals inferred from brain transcriptomes using the coalescent-based approach.

Fig. S18. Topological distances (d_T) between the mammalian PG tree and brain PT_{HaMStR} trees when the mammalian (A) or eukaryotic (B) core orthologs are used in HaMStR ortholog identification, respectively, and topological distances (d_T) between the plant PG tree and leaf PT_{HaMStR} trees when the plant (C) or eukaryotic (D) core orthologs are used in HaMStR ortholog identification, respectively. Arrow indicates d_T based on the original data, whereas the grey shade shows the distribution of d_T based on 200 bootstrap PT trees.

Fig. S19. Summary statistics of the BUSCO analysis for each transcriptome of (A) mammals or (B) plants. Complete, duplicated, fragmented, and missing genes are indicated by dark blue, blue, orange, and red colors, respectively.

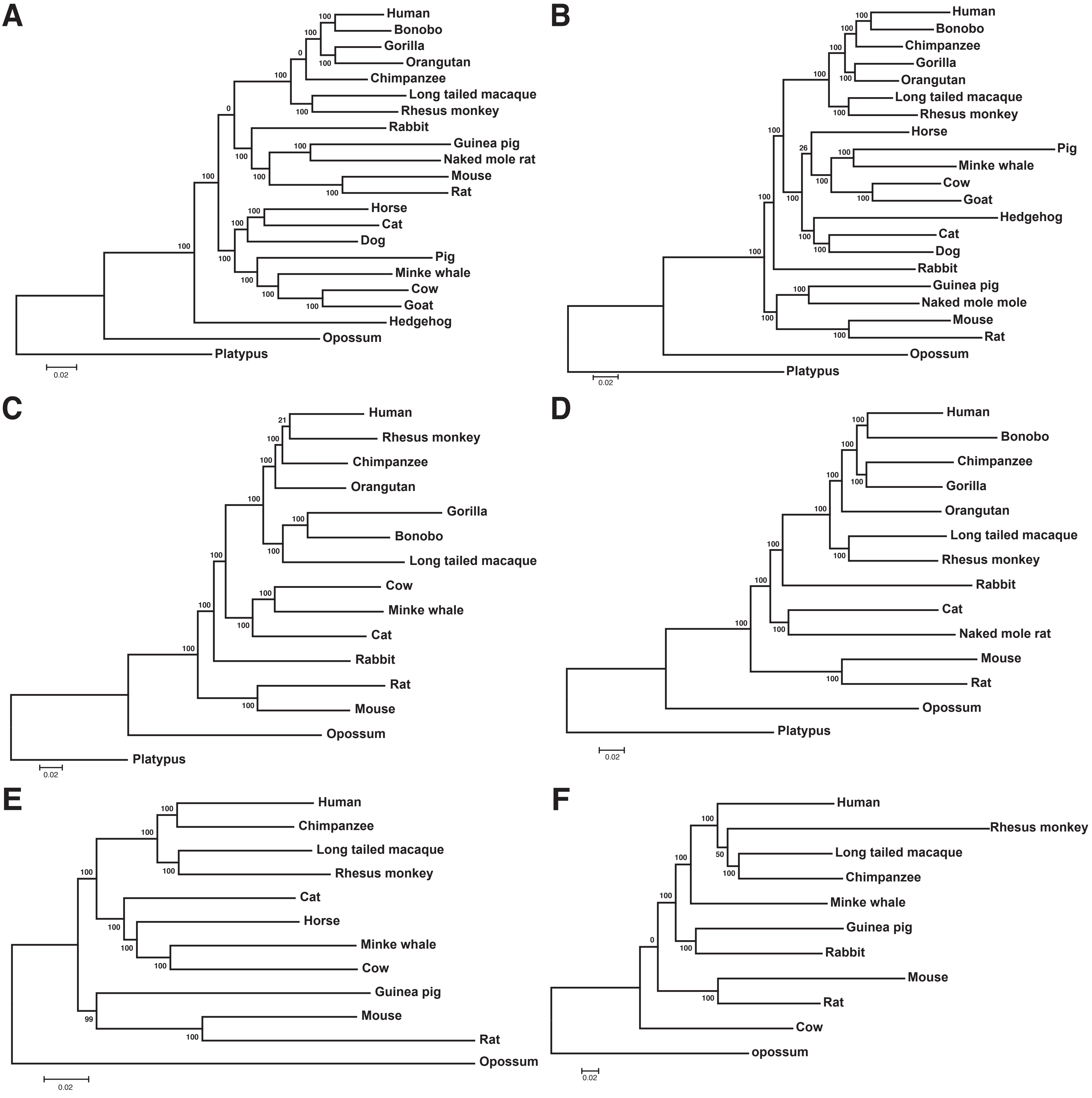

Supplemental figure 1

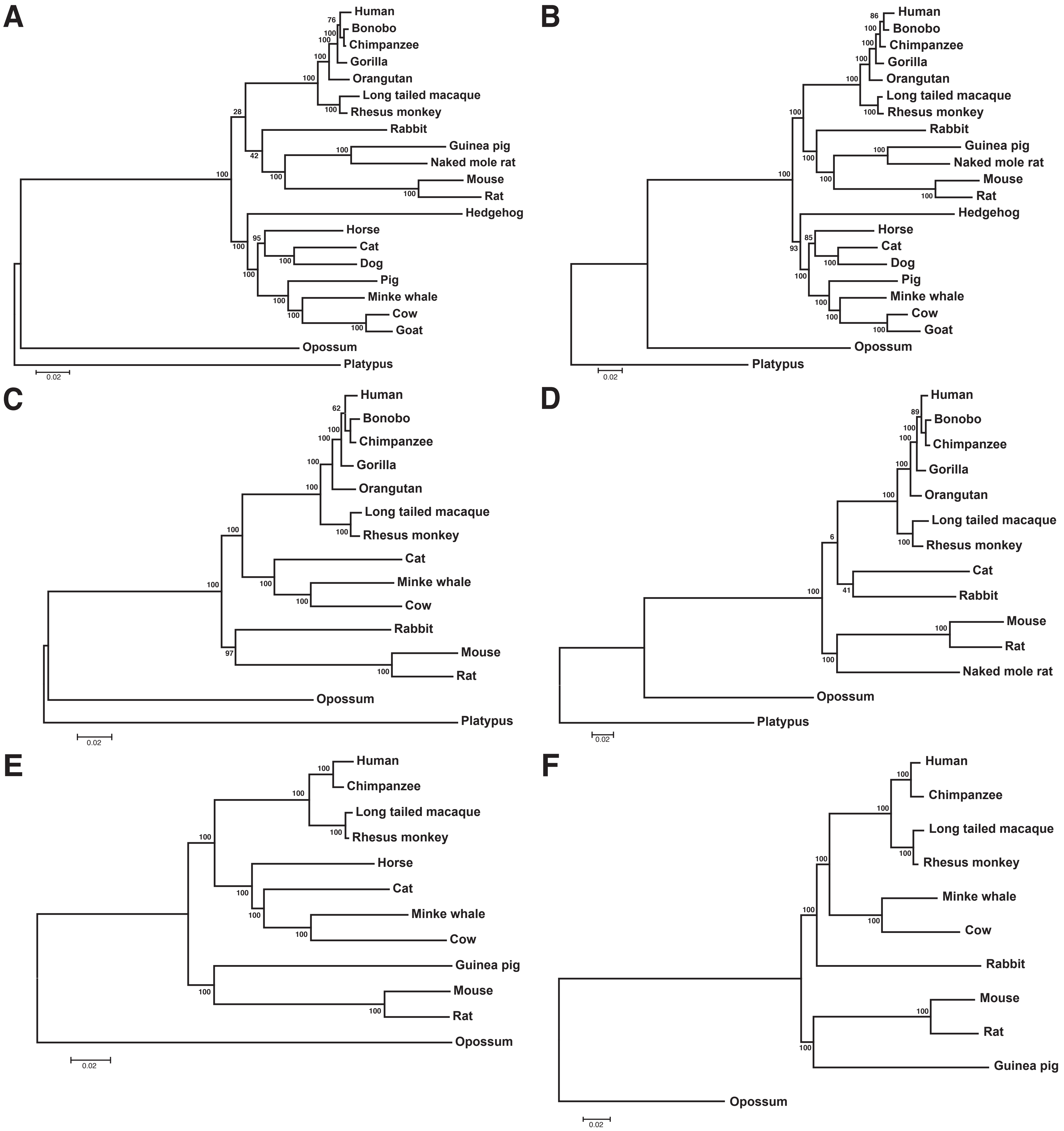

Supplemental figure 2

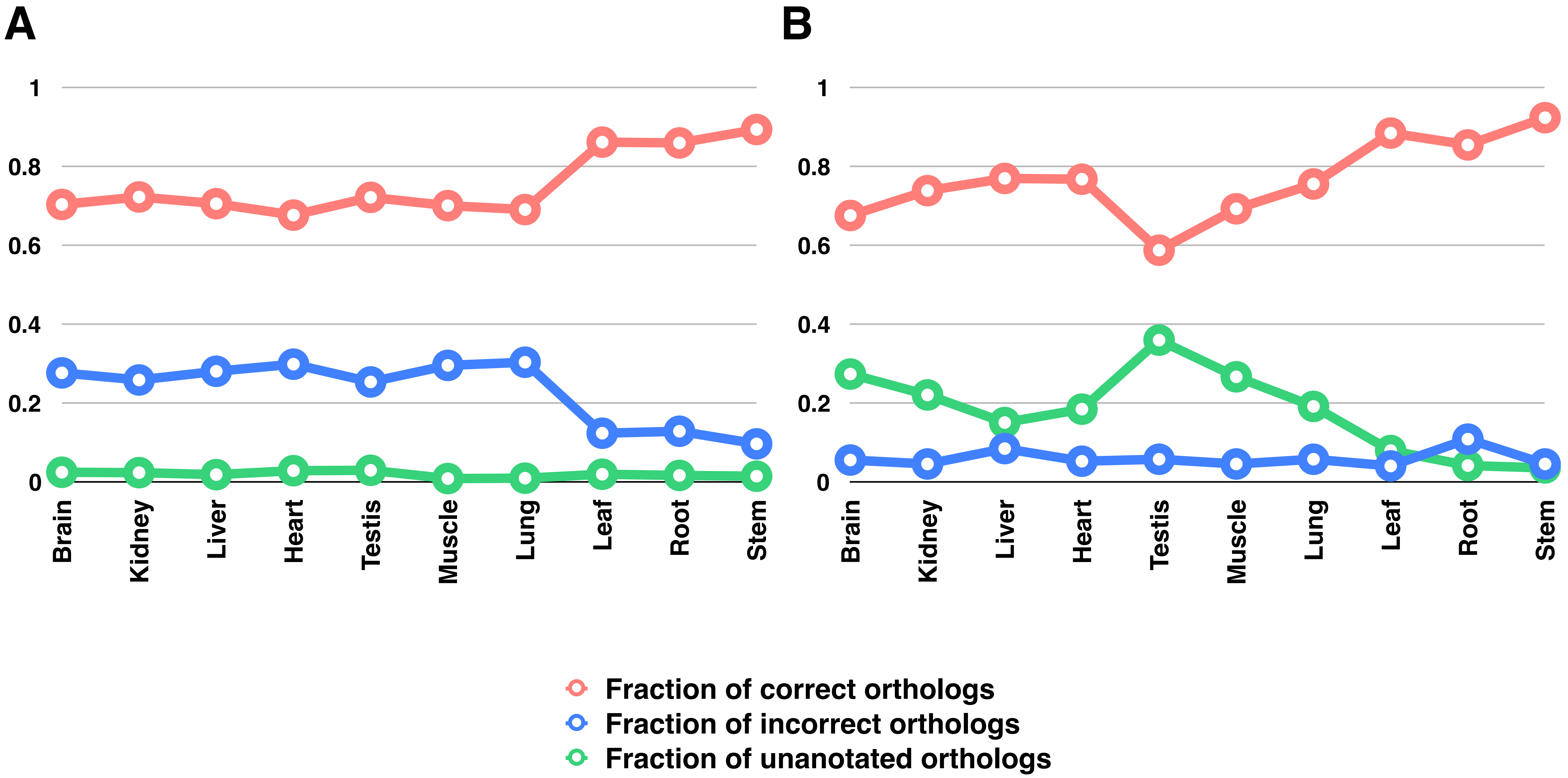

Supplemental figure 3

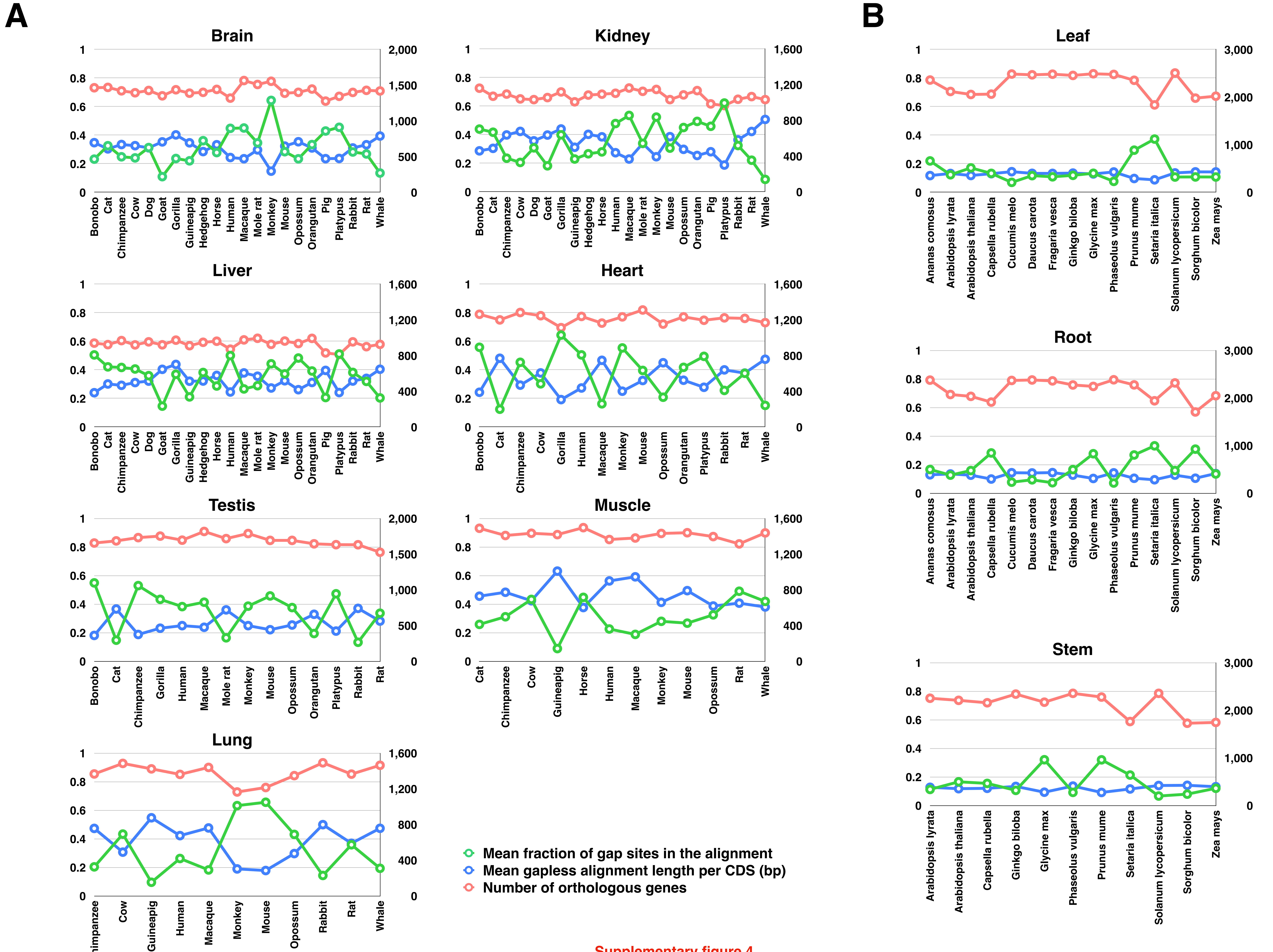

Supplementary figure 4

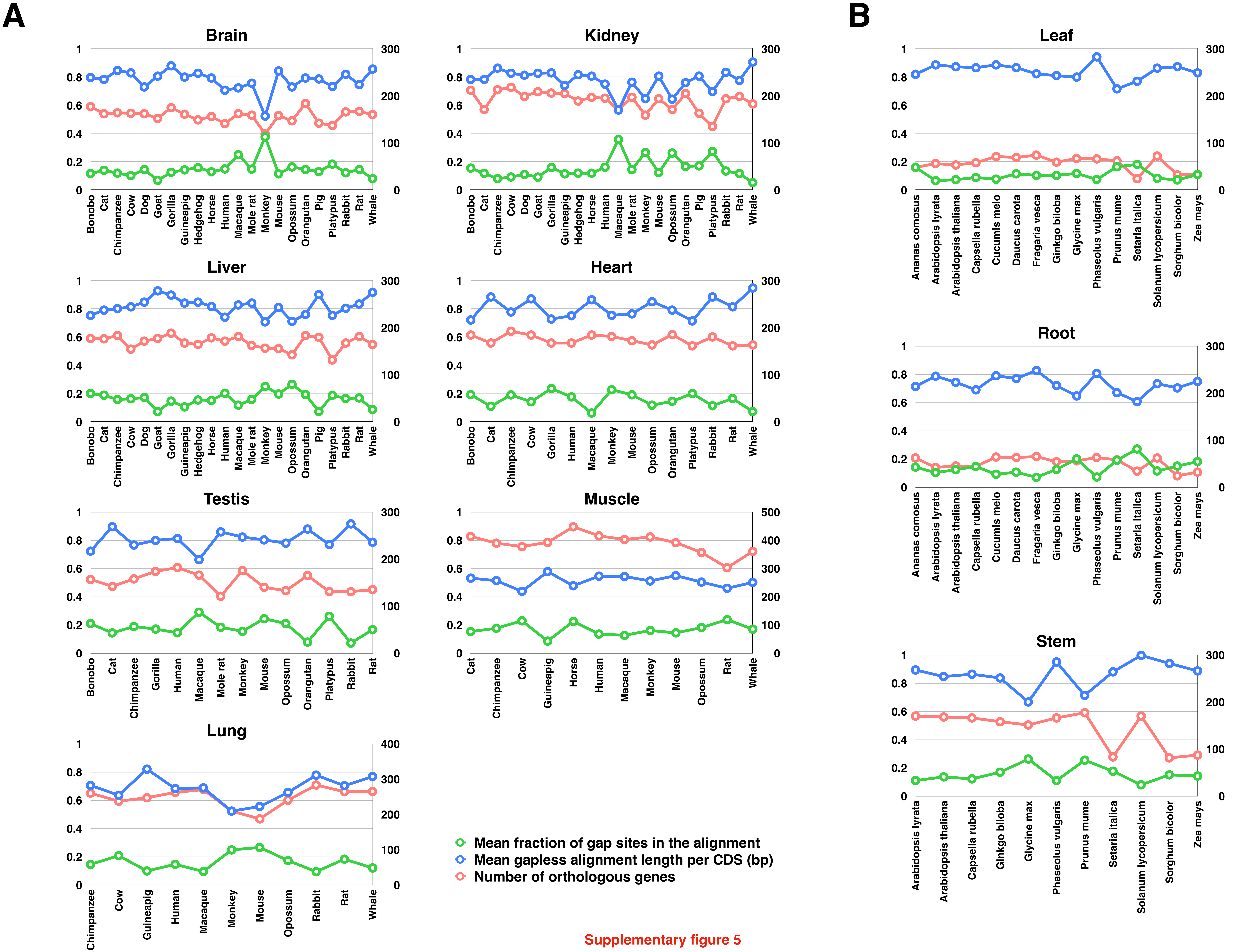

Supplementary figure 5

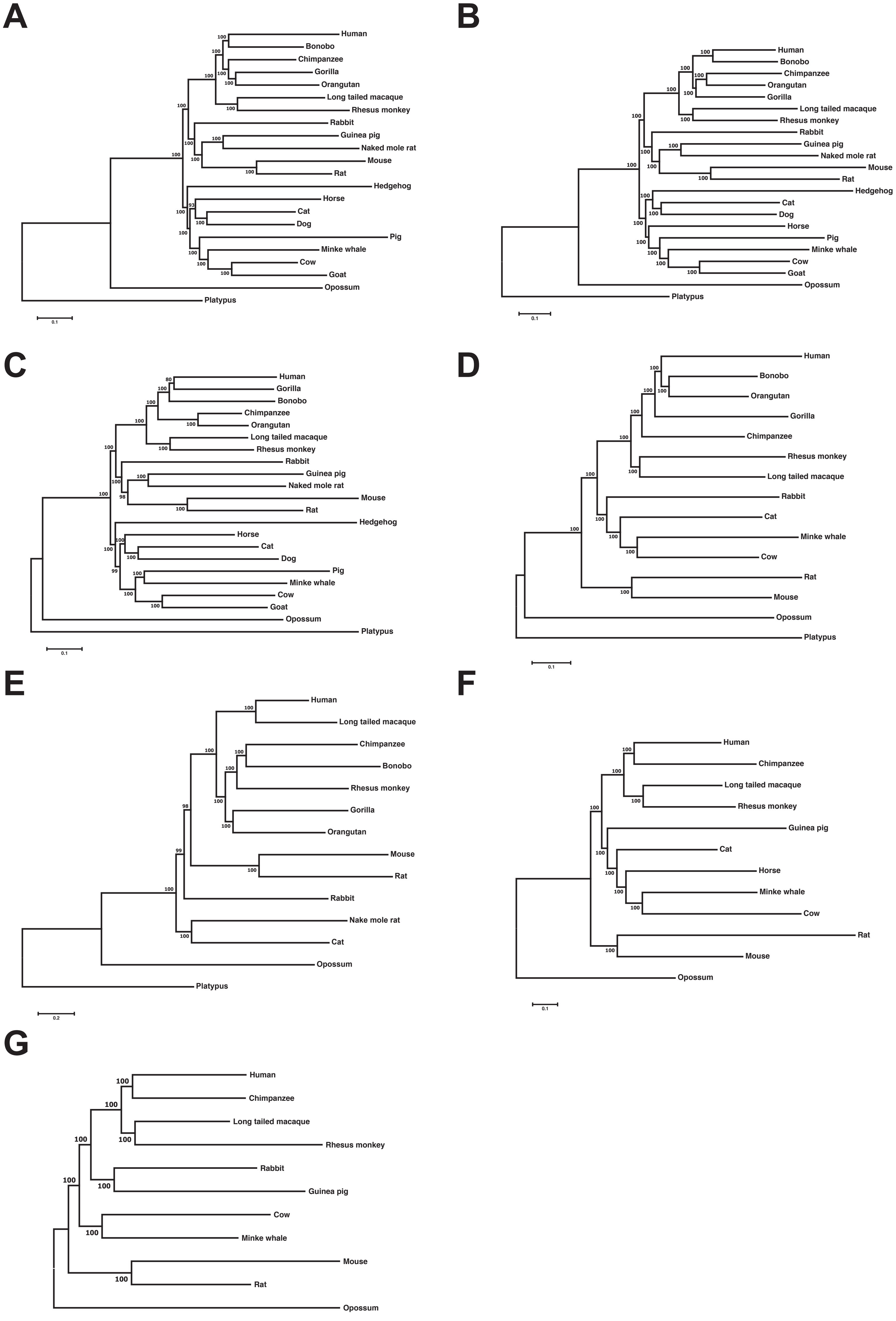

Supplemental figure 6

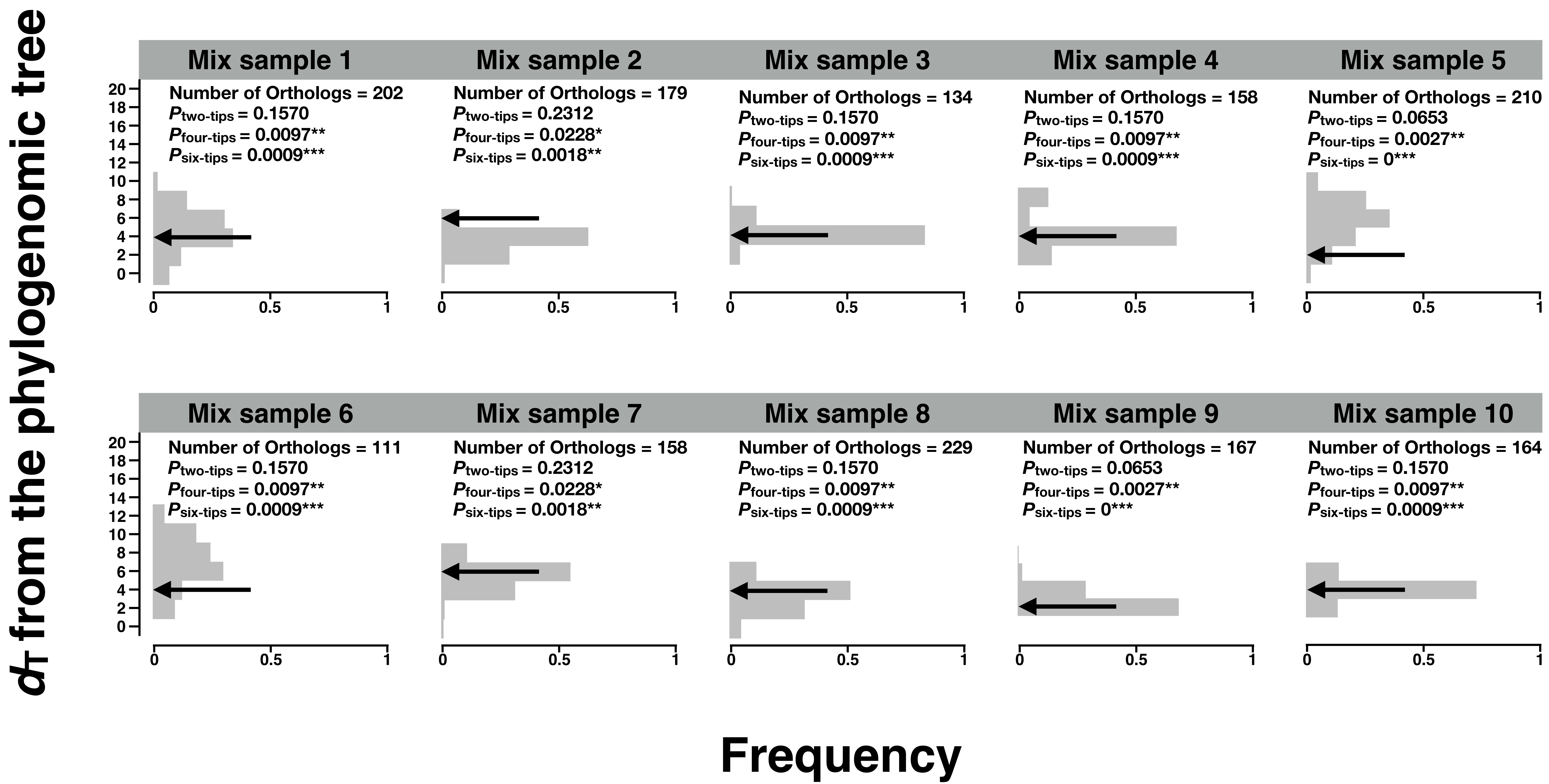

Supplemental figure 7

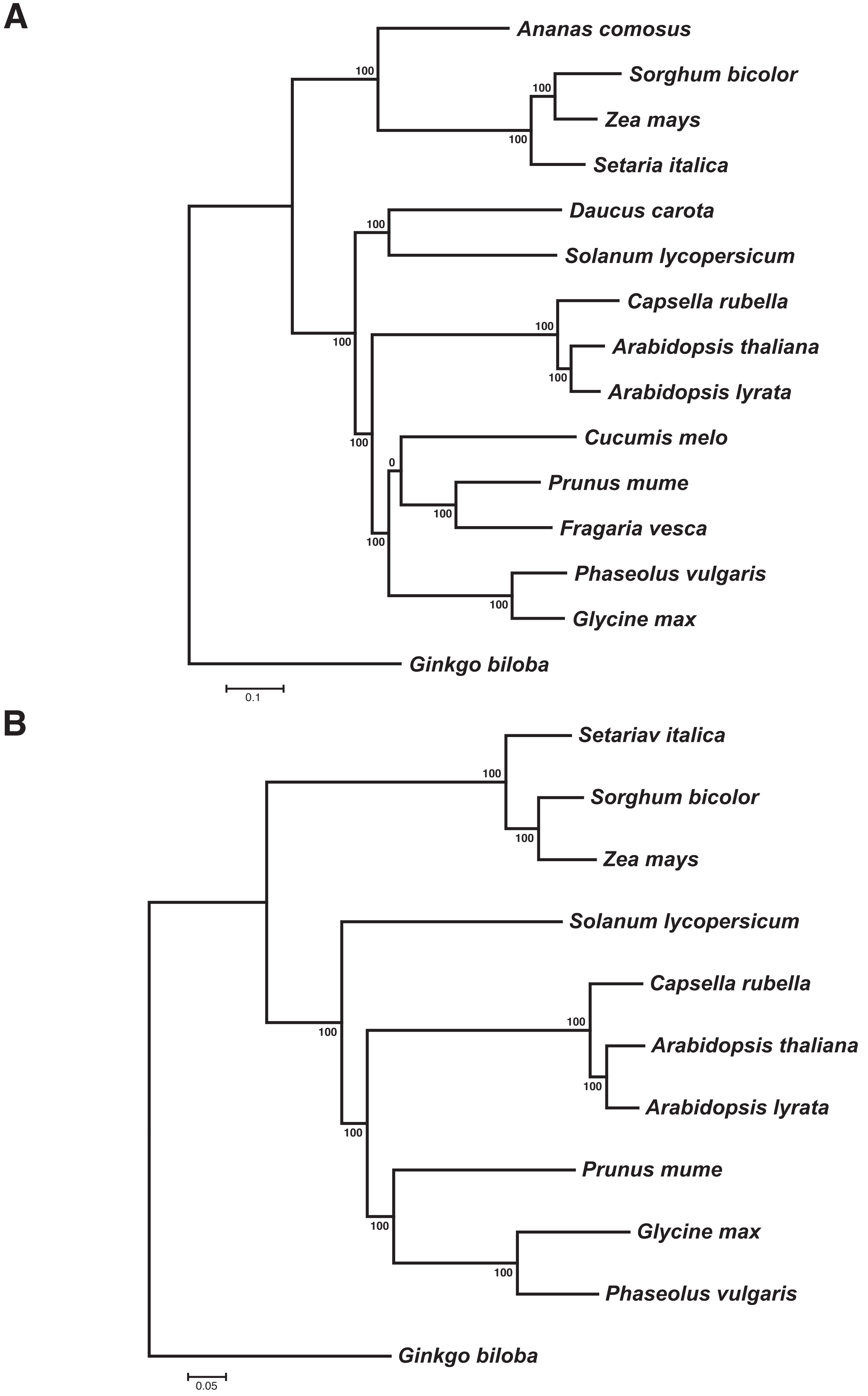

Supplementary figure 8

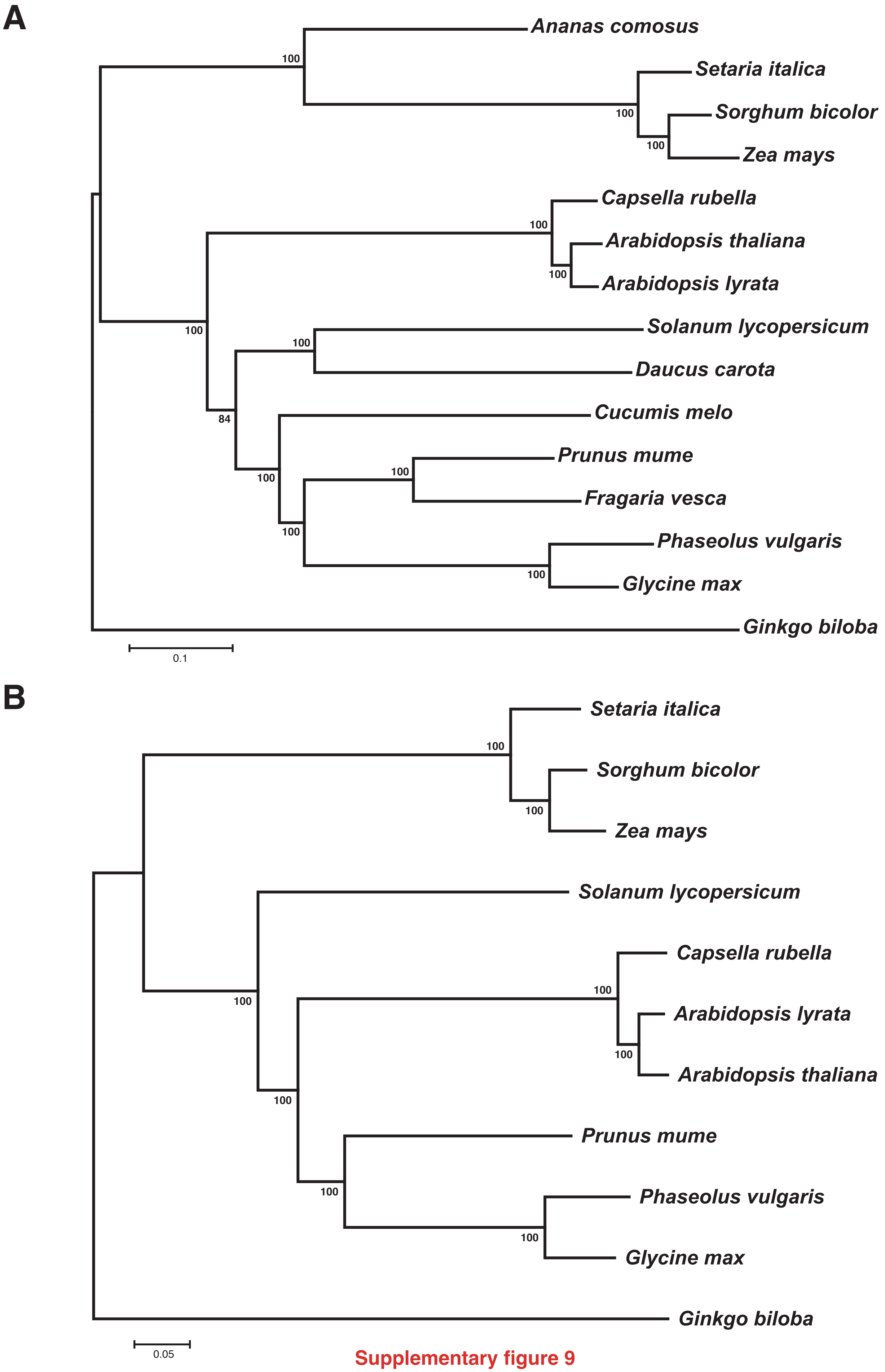

Supplementary figure 9

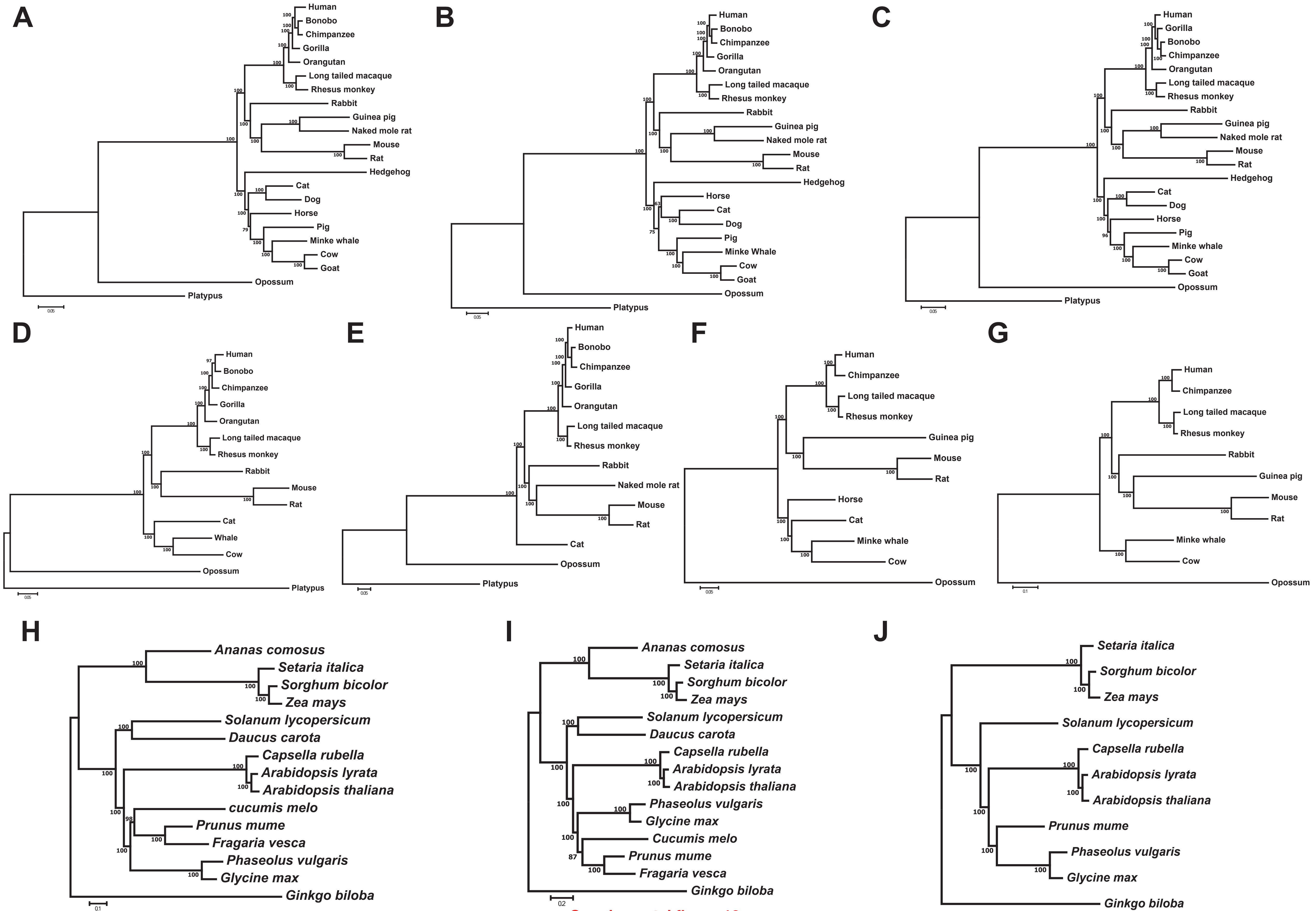

Supplemental figure 10

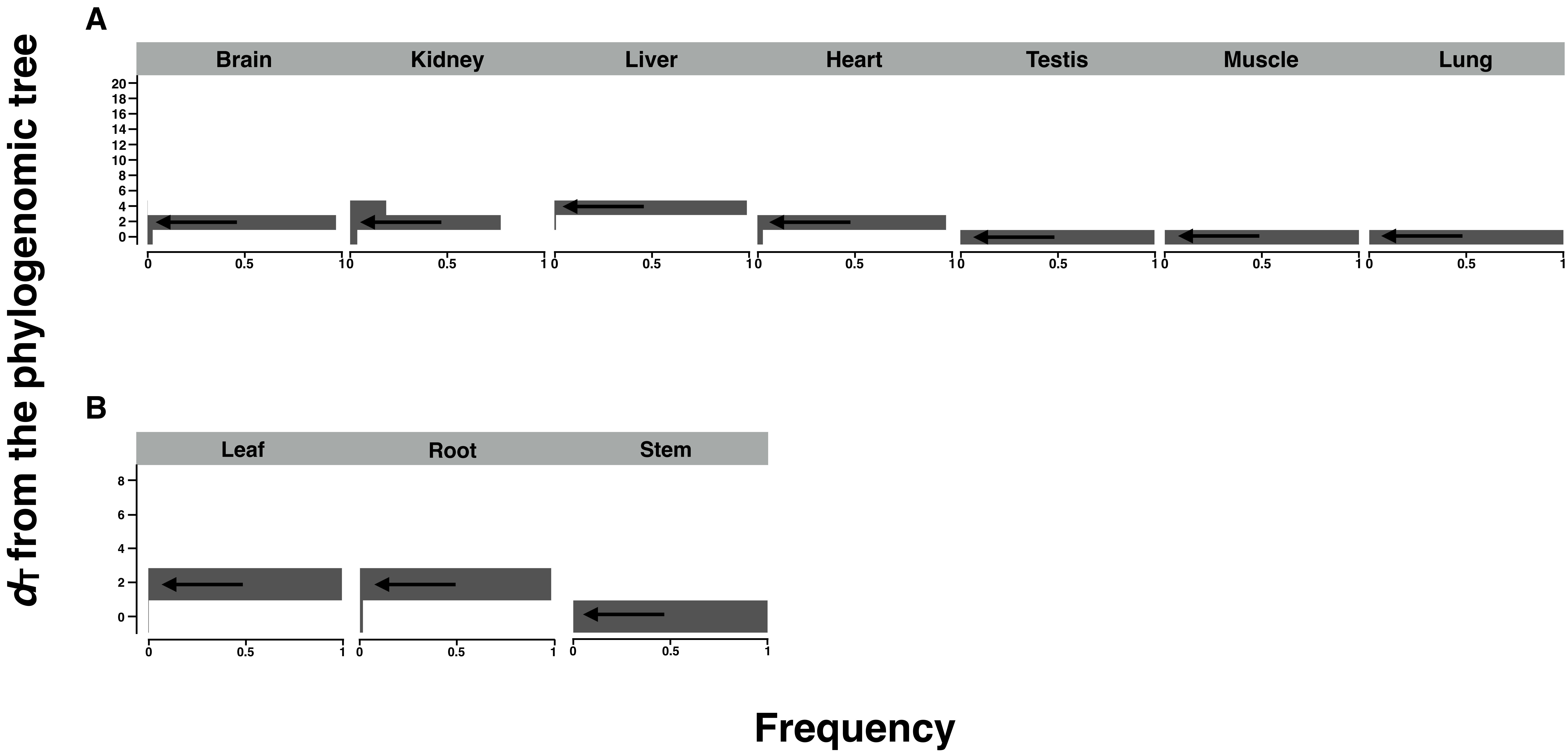

Supplemental figure 11

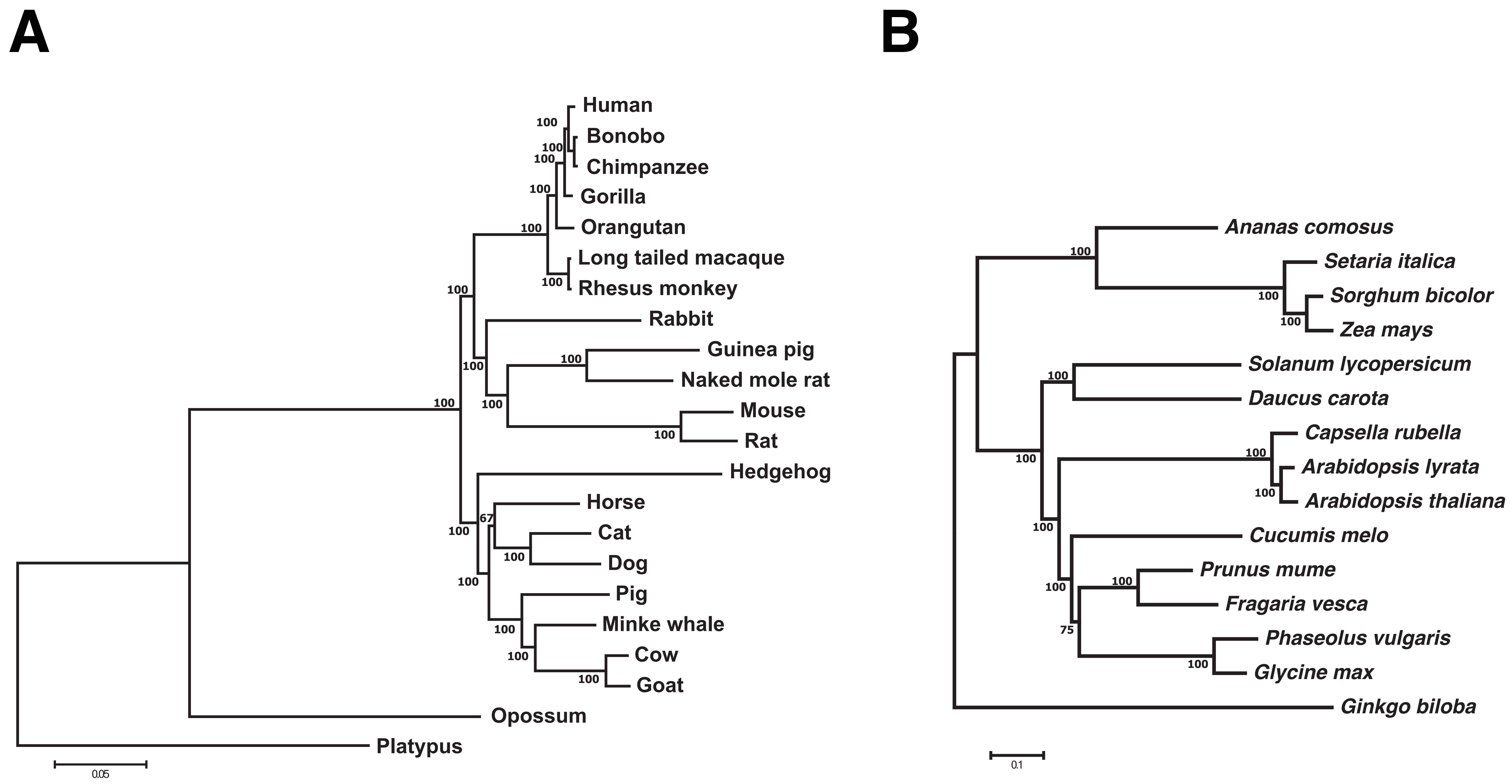

Supplementary figure 12

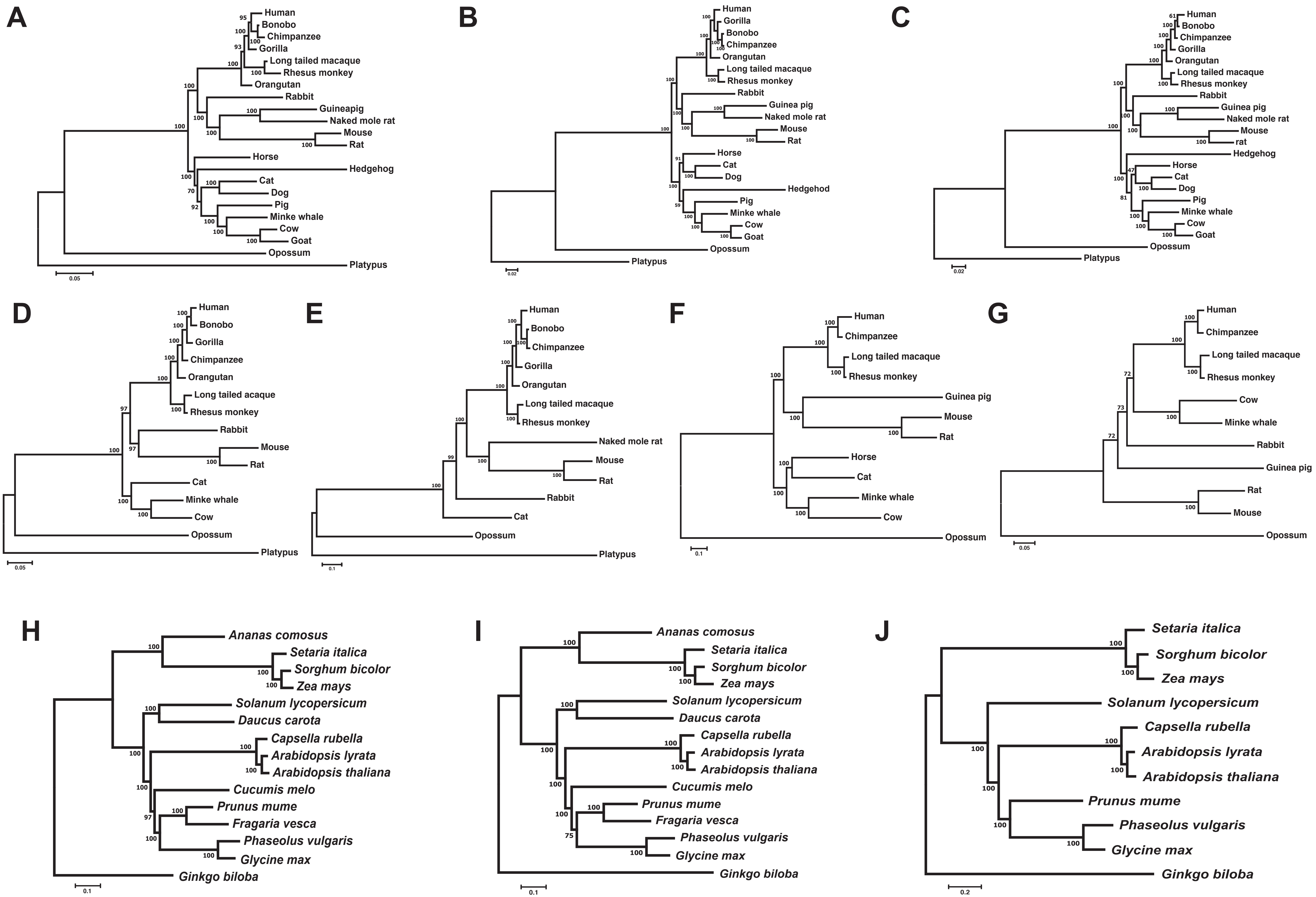

Supplemental figure 13

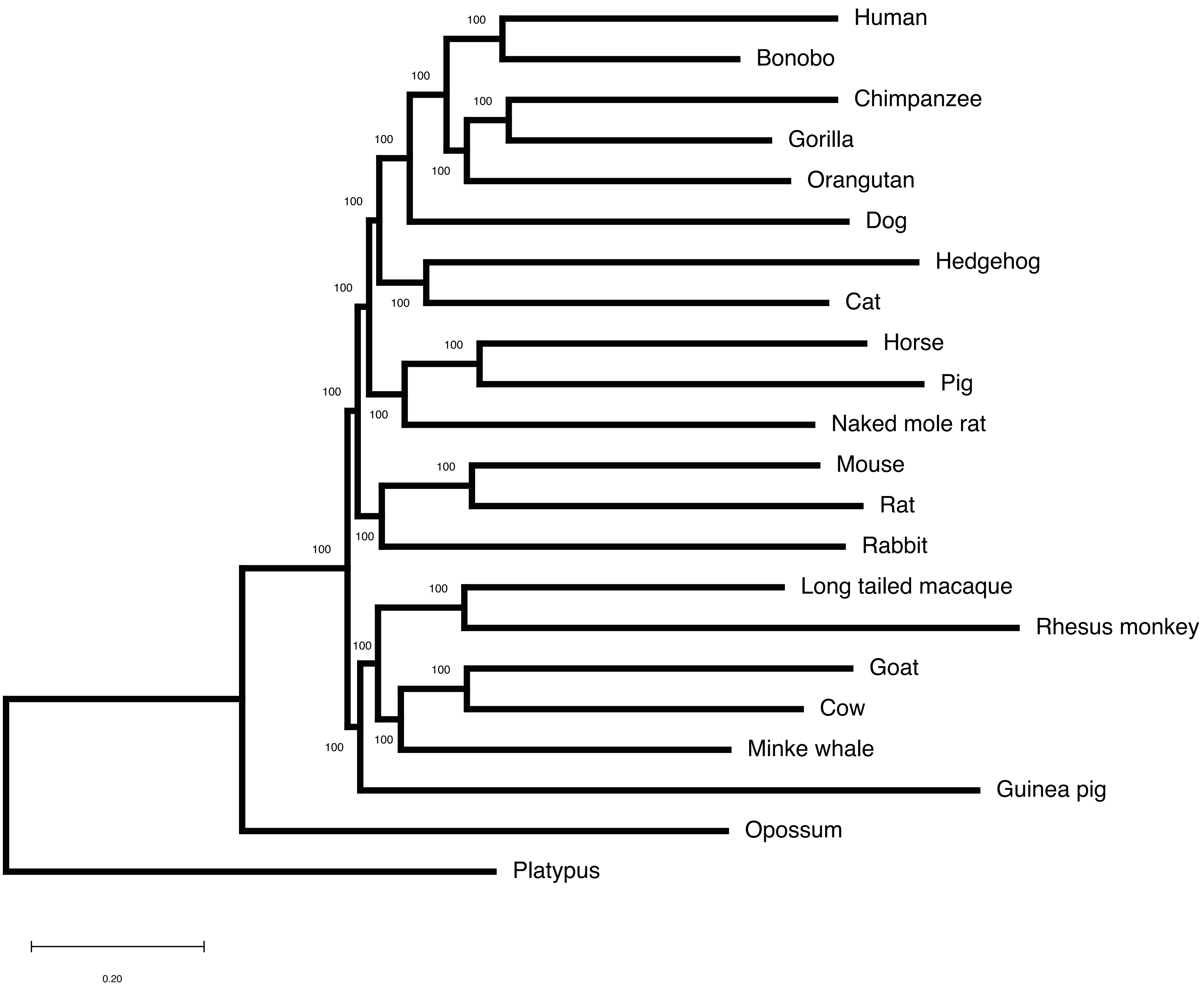

Supplemental figure 14

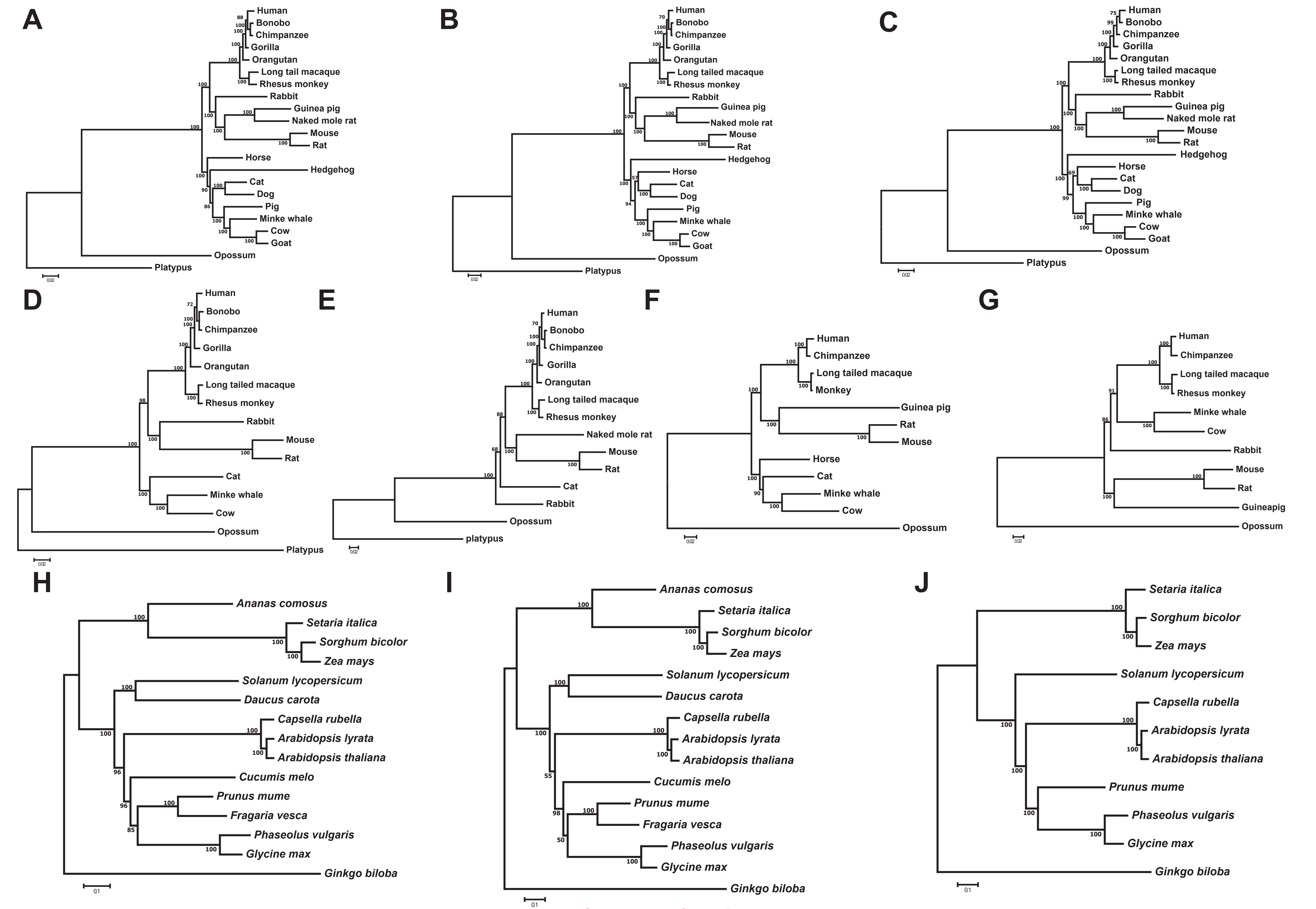

Supplemental figure 15

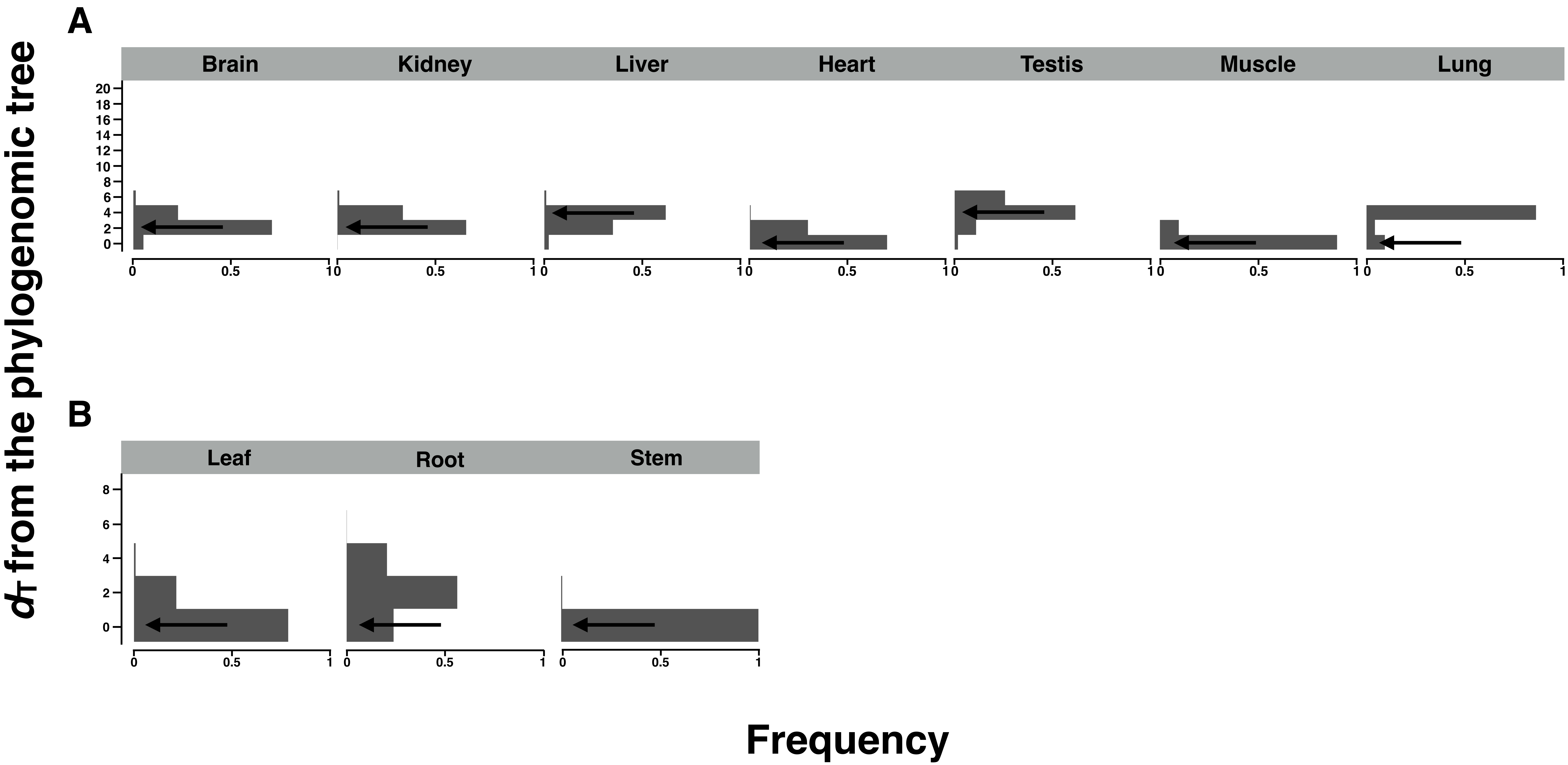

Supplemental figure 16

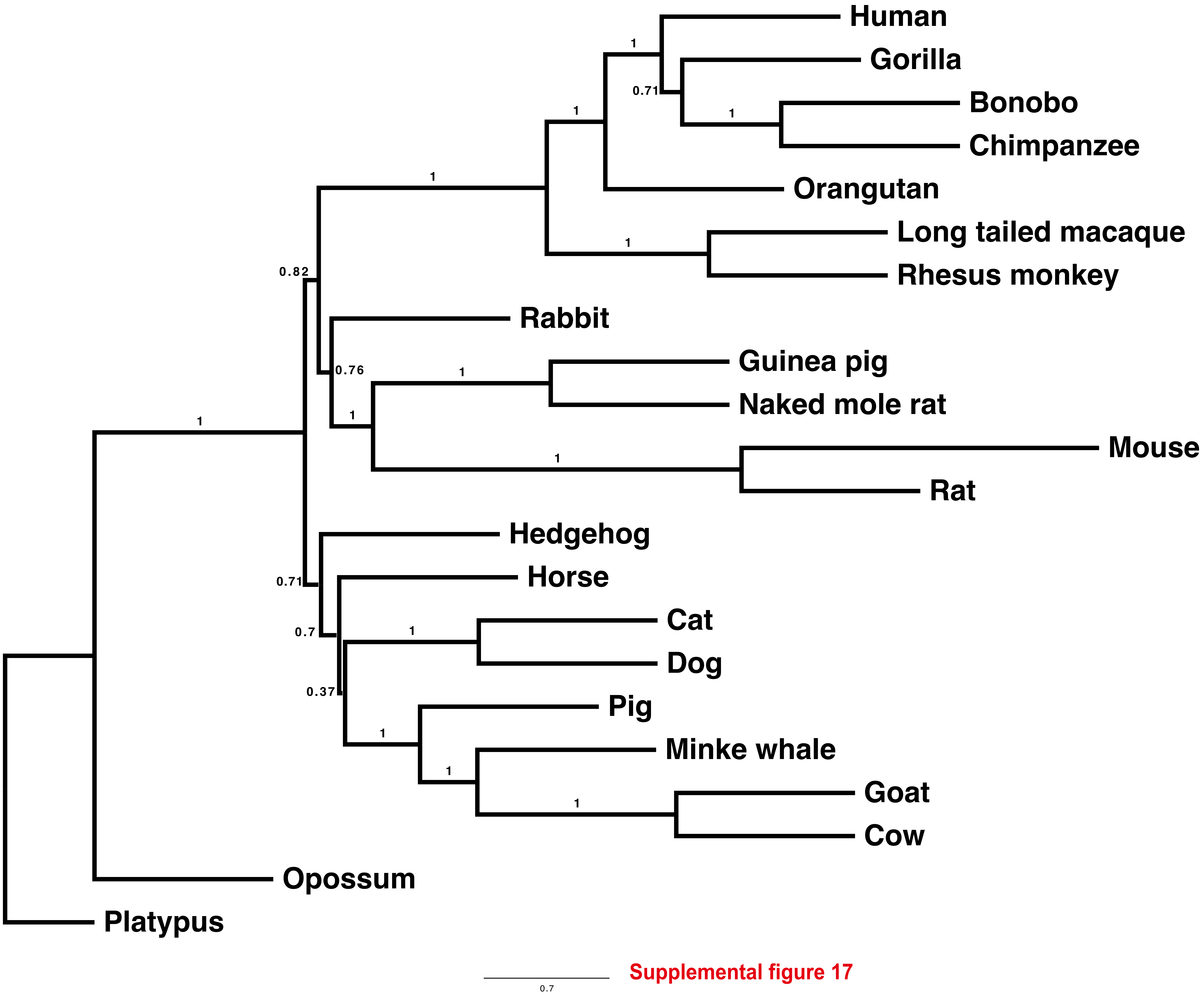

Supplemental figure 17

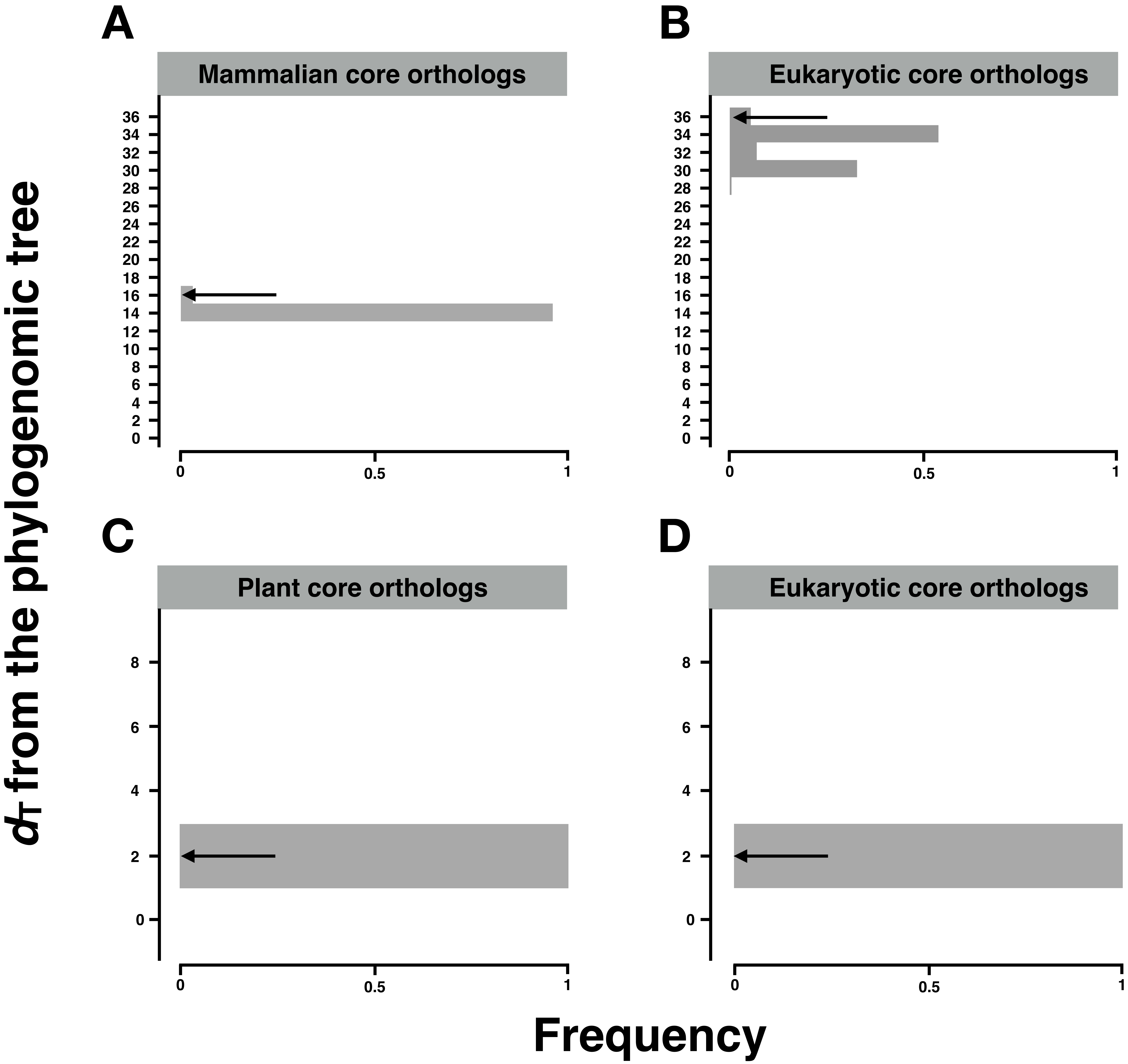

Supplemental Figure 18

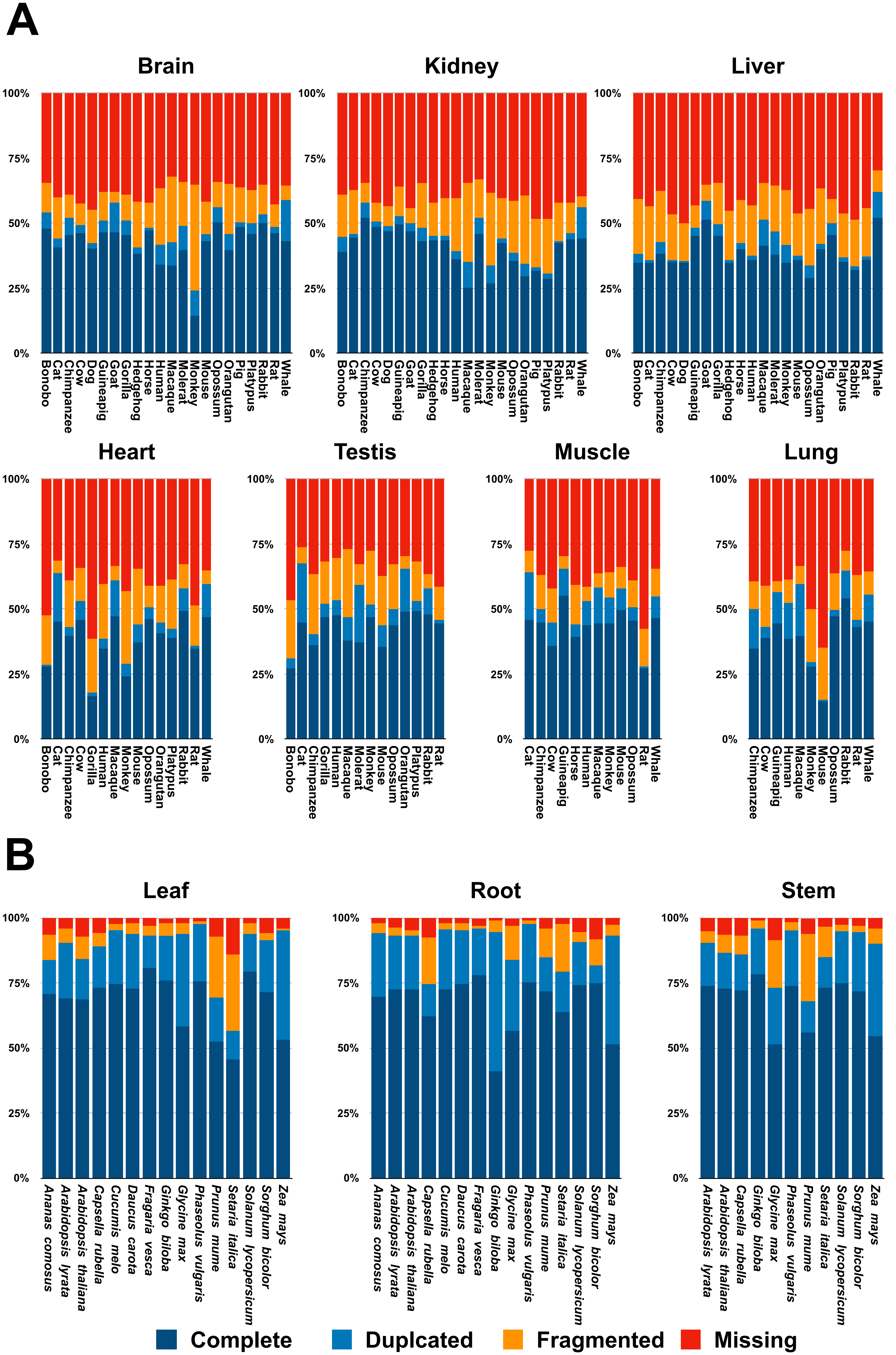

Supplementary figure 19

Table S1. Data used for the 22 mammals

Common name	Species name	Genome-based protein sequences		Transcriptome raw data				
		Database	Version	Tissue	Bioproject	SRA No.	Library	Data size (MB)
Bonobo	*Pan paniscus*	NCBI	panpan1.1	Brain	PRJNA143627	SRR306826	PAIRED	1733
				Heart	PRJNA143627	SRR306831	SINGLE	1731
				kidney	PRJNA143627	SRR306833	SINGLE	2640
				Liver	PRJNA143627	SRR306835	SINGLE	1644
				Testis	PRJNA143627	SRR306837	SINGLE	1056
Cat	*Felis catus*	NCBI	Felis_catus_8.0	Brain	PRJNA184055	SRR636949	PAIRED	1647
				Heart	PRJNA312519	SRR3200471	PAIRED	9865
				kidney	PRJNA184055	SRR636905	PAIRED	1476
				Liver	PRJNA184055	SRR636856	PAIRED	1480
				Muscle	PRJNA312519	SRR3200451	PAIRED	5020
				Testis	PRJNA312519	SRR3200462	PAIRED	7352
Chimpanzee	*Pan troglodytes*	NCBI	Pan_tro 3.0	Brain	PRJNA143627	SRR306815	PAIRED	1972
				Heart	PRJNA143627	SRR306820	SINGLE	2478
				kidney	PRJNA143627	SRR306822	SINGLE	3403
				Liver	PRJNA143627	SRR306823	SINGLE	1739
				Lung	PRJNA271912	SRR1758926	PAIRED	5126
				Muscle	PRJNA271912	SRR1758930	PAIRED	2484
				Testis	PRJNA143627	SRR306825	SINGLE	1511
Cow	*Bos taurus*	NCBI	Bos_taurus_UMD_3.1.1	Brain	PRJNA184055	SRR636934	PAIRED	1725
				Heart	PRJNA251439	SRR1449265	PAIRED	3139
				kidney	PRJNA184055	SRR636890	PAIRED	1700
				Liver	PRJNA184055	SRR636839	PAIRED	1039
				Lung	PRJNA251439	SRR1449271	PAIRED	2983
				Muscle	PRJNA251439	SRR1449282	PAIRED	1759
Dog	*Canis lupus familiaris*	NCBI	CanFam3.1	Brain	PRJNA184055	SRR636937	PAIRED	939
				kidney	PRJNA184055	SRR636892	PAIRED	1260
				Liver	PRJNA184055	SRR636843	PAIRED	1076
Goat	*Capra hircus*	NCBI	ARS1	Brain	PRJNA184055	SRR636940	PAIRED	3383
				kidney	PRJNA184055	SRR636895	PAIRED	2493
				Liver	PRJNA184055	SRR636845	PAIRED	2127

Gorilla	*Gorilla gorilla*	NCBI	gorGor4	Brain	PRJNA143627	SRR306801	PAIRED	1715
				Heart	PRJNA143627	SRR306805	SINGLE	1725
				kidney	PRJNA143627	SRR306807	SINGLE	2496
				Liver	PRJNA143627	SRR306809	SINGLE	2015
				Testis	PRJNA143627	SRR306810	SINGLE	1196
Guinea pig	*Cavia porcellus*	NCBI	Cavpor3.0	Brain	PRJNA184055	SRR636941	PAIRED	1724
				kidney	PRJNA184055	SRR636896	PAIRED	1497
				Liver	PRJNA184055	SRR636847	PAIRED	2196
				Lung	PRJNA242409	SRR1200908	PAIRED	8078
				Muscle	PRJNA242409	SRR1200910	PAIRED	16228
Hedgehog	*Erinaceus europaeus*	NCBI	EriEur2.0	Brain	PRJNA184055	SRR636947	PAIRED	1637
				kidney	PRJNA184055	SRR636902	PAIRED	1517
				Liver	PRJNA184055	SRR636852	PAIRED	1628
Horse	*Equus caballus*	NCBI	Ajinai1.0	Brain	PRJNA184055	SRR636944	PAIRED	1529
				kidney	PRJNA184055	SRR636901	PAIRED	1021
				Liver	PRJNA184055	SRR636849	PAIRED	1521
				Muscle	PRJNA164679	SRR495388	PAIRED	2938
Human	*Homo sapiens*	NCBI	GRCh38	Brain	PRJNA143627	SRR306842	PAIRED	1780
				Heart	PRJNA143627	SRR306850	SINGLE	1635
				kidney	PRJNA143627	SRR306853	SINGLE	2669
				Liver	PRJNA143627	SRR306855	SINGLE	1494
				Lung	PRJNA280600	SRR1957195	PAIRED	4016
				Muscle	PRJNA280600	SRR1957201	PAIRED	4651
				Testis	PRJNA143627	SRR306858	SINGLE	1853
long tailed macaque	*Macaca fascicularis*	NCBI	Macaca_fascicularis_5.0	Brain	PRJNA141257	SRR223512	PAIRED	3665
				Heart	PRJNA271912	SRR1758954	PAIRED	8125
				kidney	PRJNA141257	SRR223514	PAIRED	3585
				Liver	PRJNA141257	SRR223515	PAIRED	3434
				Lung	PRJNA271912	SRR1758943	PAIRED	9279
				Muscle	PRJNA271912	SRR1758959	PAIRED	9470
				Testis	PRJNA141257	SRR223516	PAIRED	3662
Minke whale	*Balaenoptera acutorostrata*	NCBI	BalAcu1.0	Brain	PRJNA72723	SRR918699	PAIRED	3821
				Heart	PRJNA72723	SRR918701	PAIRED	5721
				kidney	PRJNA72723	SRR919295	PAIRED	3410

				Liver	PRJNA72723	SRR919296	PAIRED	3159
				Lung	PRJNA72723	SRR922125	PAIRED	2000
				Muscle	PRJNA72723	SRR923807	PAIRED	2068
Mouse	*Mus musculus*	NCBI	GRCm38	Brain	PRJNA184055	SRR636963	PAIRED	1330
				Heart	PRJNA143627	SRR306766	SINGLE	2498
				kidney	PRJNA184055	SRR636916	PAIRED	1056
				Liver	PRJNA184055	SRR636869	PAIRED	1079
				Lung	PRJEB11897	ERR1146828	PAIRED	2080
				Muscle	PRJEB11897	ERR1146833	PAIRED	2122
				Testis	PRJNA143627	SRR306776	SINGLE	1705
Naked mole rat	*Heterocephalus glaber*	NCBI	HetGla_female_1.0	Brain	PRJNA143625	SRR306402	PAIRED	2826
				kidney	PRJNA143625	SRR306399	PAIRED	2777
				Liver	PRJNA143625	SRR306395	PAIRED	2985
				Testis	PRJNA283581	SRR2120769	PAIRED	4628
Orangutan	*Pongo pygmaeus*	NCBI	NGS12	Brain	PRJNA143627	SRR306792	PAIRED	1746
				Heart	PRJNA143627	SRR306794	SINGLE	2105
				kidney	PRJNA143627	SRR306796	SINGLE	2713
				Liver	PRJNA143627	SRR306799	SINGLE	2042
				Testis	PRJNA293451	SRR2176207	PAIRED	3674
Pig	*Sus scrofa*	NCBI	Sscrofa11	Brain	PRJNA184055	SRR636929	PAIRED	1086
				kidney	PRJNA184055	SRR636881	PAIRED	1002
				Liver	PRJNA184055	SRR636974	PAIRED	2392
Platypus	*Ornithorhynchus anatinus*	NCBI	Ornithorhynchus_anatinus-5.0.1	Brain	PRJNA143627	SRR306725	SINGLE	982
				Heart	PRJNA143627	SRR306730	SINGLE	1089
				kidney	PRJNA143627	SRR306732	SINGLE	1436
				Liver	PRJNA143627	SRR306736	SINGLE	1264
				Testis	PRJNA143627	SRR306741	SINGLE	1496
Rabbit	*Oryctolagus cuniculus*	NCBI	OryCun2.0	Brain	PRJNA184055	SRR636964	PAIRED	1691
				Heart	PRJNA274427	SRR1789062	PAIRED	3549
				kidney	PRJNA184055	SRR636920	PAIRED	891
				Liver	PRJNA184055	SRR636871	PAIRED	1091
				Lung	PRJNA78323	SRR388301	PAIRED	2314
				Testis	PRJNA78323	SRR388292	PAIRED	2915
Rat	*Rattus norvegicus*	NCBI	Rnor_6.0	Brain	PRJNA184055	SRR636970	PAIRED	1087

				Heart	PRJNA238328	SRR1170039	SINGLE	1057
				kidney	PRJNA184055	SRR636926	PAIRED	1277
				Liver	PRJNA184055	SRR636875	PAIRED	1359
				Lung	PRJNA238328	SRR1170181	SINGLE	1009
				Muscle	PRJNA238328	SRR1170302	SINGLE	805
				Testis	PRJNA238328	SRR1170500	SINGLE	1130
Rhesus monkey	*Macaca mulatta*	NCBI	Mmul_8.0.1	Brain	PRJNA141257	SRR223518	PAIRED	2410
				Heart	PRJNA143627	SRR306782	SINGLE	1625
				kidney	PRJNA141257	SRR223520	PAIRED	2679
				Liver	PRJNA141257	SRR223521	PAIRED	2891
				Lung	PRJNA230664	SRR1047643	SINGLE	1753
				Muscle	PRJNA149557	SRR389102	PAIRED	6946
				Testis	PRJNA143627	SRR306790	SINGLE	1862
Short tailed opossum	*Monodelphis domestica*	NCBI	MonDom5	Brain	PRJNA143627	SRR306743	SINGLE	2702
				Heart	PRJNA164819	SRR500904	PAIRED	3923
				kidney	PRJNA143627	SRR306751	SINGLE	1221
				Liver	PRJNA143627	SRR306753	SINGLE	1159
				Lung	PRJNA164819	SRR500897	PAIRED	3873
				Muscle	PRJNA164819	SRR500912	PAIRED	3465
				Testis	PRJNA143627	SRR306756	SINGLE	1927

Table S2. Mean number of excluded species per orthologous gene in various transcriptome data

Tissue	Number of species	Mean number of excluded species per orthologous gene		
		HaMStR	YS	*P* -value*
Brain	22	6.596	9.584	< 2.2e-16
Kidney	22	6.556	9.621	< 2.2e-16
Liver	22	6.785	9.725	< 2.2e-16
Heart	15	3.956	6.146	< 2.2e-16
Testis	14	3.706	6.123	< 2.2e-16
Muscle	12	3.584	5.137	< 2.2e-16
Lung	11	3.090	4.370	< 2.2e-16
Leaf	15	1.889	4.039	2.85E-14
Root	15	1.945	4.760	< 2.2e-16
Stem	12	2.478	4.270	< 2.2e-16

*Mann-Whitney U test comparing excluded species numbers of individual genes between HaMStR and YS.

Table S3. Data used for the 15 plants

Species name	Genome-based protein sequences		Transcriptome raw data				
	Database	Version	Tissue	Bioproject	SRA No.	Library	Data size (MB)
Arabidopsis lyrata	NCBI	v.1.0	leaf	PRJNA336053	SRR3993764	PAIRED	1621
			root		SRR3993767	PAIRED	1306
			stem		SRR3993766	PAIRED	1311
Arabidopsis thaliana	NCBI	TAIR10	leaf	PRJNA336053	SRR3993754	PAIRED	769
			root		SRR3993762	PAIRED	856
			stem		SRR3993761	PAIRED	717
Capsella rubella	NCBI	Caprub1_0	leaf	PRJNA336053	SRR3993756	PAIRED	968
			root		SRR3993759	PAIRED	729
			stem		SRR3993758	PAIRED	842
Setaria italica	NCBI	Setaria_italica_v2.0	leaf	PRJNA153279	SRR442162	PAIRED	1987.57
			root		SRR442161	PAIRED	1961
			stem		SRR442163	PAIRED	1968.36
Zea mays	NCBI	ZmaysB73_wgs_1.0	leaf	PRJNA291064	SRR2129982	PAIRED	2829.24
			root		SRR2129986	PAIRED	3051.81
			stem		SRR2129984	PAIRED	2489.06
Glycine max	NCBI	Glycine_max_v2.0	leaf	PRJNA140081	SRR203363	SINGLE	1988.84
			root		SRR203364	SINGLE	1731.88
			stem		SRR203365	SINGLE	738.88
Prunus mume	NCBI	P.mume_V1.0	leaf	PRJNA172987	SRR542479	PAIRED	1340.66
			root		SRR542480	PAIRED	1649.85
			stem		SRR542481	PAIRED	1058.02
Solanum lycopersicum	NCBI	V100	leaf	PRJNA307656	SRR3095793	SINGLE	1288.81
			root		SRR3095831	SINGLE	1370.53
			stem	PRJNA271503	SRR2239884	PAIRED	2834.8
Phaseolus vulgaris	NCBI	PhaVulg1_0	leaf	PRJNA359576	SRR5137647	PAIRED	6816.15
			root		SRR5137652	PAIRED	8523.54
			stem	PRJNA221782	SRR1025294	PAIRED	2949.99
Sorghum bicolor	NCBI	Sorghum_bicolor_NCBLv3	leaf	PRJDB4770	DRR059875	PAIRED	2886.05

			stem		DRR059876	PAIRED	2646.85
			root	PRJNA143783	SRR299235	SINGLE	859.89
Daucus carota	NCBI	ASM162521v1	leaf	PRJNA391808	SRR5829255	SINGLE	1772.45
			root		SRR5829254	SINGLE	1862.09
Fragaria vesca	NCBI	FraVesHawaii_1.0	leaf	PRJNA272956	SRR1930097	PAIRED	4346
			root	PRJNA327720	SRR3743193	PAIRED	6124.7
Ananas comosus	NCBI	ASM154086v1	leaf	PRJNA305042	SRR2976088	SINGLE	1092.81
			root		SRR2976057	SINGLE	1569.93
Cucumis melo	NCBI	ASM31304v1	leaf	PRJNA383830	SRR5465692	PAIRED	2626.51
			root		SRR5465696	PAIRED	2032.52
Ginkgo biloba	GIGADB	TM011	leaf	PRJNA80111	SRR325161	PAIRED	2208.77
			root		SRR325162	PAIRED	1978.18
			stem		SRR325166	PAIRED	2041.05
